# Structural and functional characterization of *Caenorhabditis elegans* cyclic GMP-activated channel TAX-4 via molecular dynamics simulations

**DOI:** 10.1007/s00249-025-01756-w

**Published:** 2025-05-27

**Authors:** Nicole Luchetti, Marco Lauricella, Velia Minicozzi, Grazia Cottone, Letizia Chiodo

**Affiliations:** 1 Department of Engineering, Università Campus Bio-Medico di Roma, Via Álvaro del Portillo 21, Rome, 00128 Italy; 2 Centre for Life Nano- & Neuro-Science, Italian Institute of Technology, Viale Regina Elena 291, Rome, 00161 Italy; 3 Istituto per le Applicazioni del Calcolo, Consiglio Nazionale delle Ricerche, Via dei Taurini 19, Rome, 00185 Italy; 4 Department of Physics and INFN, “Tor Vergata” University, Via della Ricerca Scientifica 1, Rome, 00133 Italy; 5 Department of Physics and Chemistry “Emilio Segrè”, University of Palermo, Viale delle Scienze 17, Palermo, 90128 Italy

**Keywords:** *C. elegans*, Ligand-gated ion channel, Molecular dynamics

## Abstract

Cyclic nucleotide-gated (CNG) ion channels are crucial to the intracellular calcium dynamics in neurons and other sensory cells, in several organisms. Mutations in CNG genes are linked to various dysfunctions and diseases. In this work, we propose a theoretical investigation of the structural and functional properties of wild-type TAX-4, a non-selective CNG ion channel, expressed in various sensory neurons of *Caenorhabditis elegans*, and involved in the permeation of monovalent and multivalent cations. Using a recent cryo-electron microscopy structure of the open state of the channel as the starting conformation, we conduct all-atom molecular dynamics simulations of the full-length channel in a membrane/water/ions system, both in the cGMP-bound and unbound conformations. Several channel structural descriptors are examined and a first-level functional annotation is carried out, on the microsecond time scale. A comparison with the available experimental data on TAX-4 and human homologues allows us to assign the simulated bound and unbound models as the pre-open and closed conformations of TAX-4, respectively. Comparisons between the bound and unbound conformations enable us to suggest key conformational changes underlying the binding-to-gating transition.

**Supplementary Information:**

The online version contains supplementary material available at 10.1007/s00249-025-01756-w.

## Introduction

The cyclic nucleotide-gated ion channels (CNGs) are non-selective cationic membrane proteins, expressed in different cells and tissues, and related to sensory transduction and hormone regulation. They belong to the superfamily of voltage-gated ion channels (Jan and Jan 1990; Kaupp and Seifert 2002), activated by cAMP (cyclic adenosine monophosphate) and cGMP (cyclic guanosine monophosphate) binding, and, to a lesser extent, by membrane voltage.

The relevance of the CNG family is related to its widespread expression and role in fundamental molecular pathways. CNG ion channels control the intracellular Ca^2+^ dynamics in neurons and other sensory cells (Kaupp and Seifert 2002; Mazzolini et al. 2018; Friebe et al. 2020; Frings et al. 1995). In mammals, a specific function of CNG is associated with photoreception. Mutations in CNG genes are associated with achromatopsia (rod monochromacy) and color blindness, which are known as cone photoreceptor disorders (CPD) (Kohl et al. 1998; Koeppen et al. 2008; Dai and Varnum 2013; Tanaka et al. 2015; Johnson et al. 2004) and retinitis pigmentosa. In addition, CNG channels regulate the rhythmic activity in the heart and brain.

In the model system *Caenorhabditis elegans* (*C. elegans*) (Li et al. 2017; Zheng et al. 2020; Nicoletti et al. 2020, 2023), TAX-4, expressed in several sensory neurons (Nicoletti et al. 2019, 2024), is the ortholog of the mammalian cyclic nucleotide-gated channel subunit alpha 3 (CNGA3), expressed in mammalian cones (Kaupp and Seifert 2002; Zheng et al. 2022). TAX-4 is involved in chemosensation and mutations in the *C. elegans* genes are responsible for thermo and chemosensation dysfunction (Komatsu et al. 1996).

TAX-4, being non-selective, allows the permeation of monovalent (Na^+^, K^+^) and divalent (Ca^2+^, Mg^2+^) cations, and also of small charged molecules (i.e., dimethylammonium − DMA) (Mazzolini et al. 2018). It shares the same tertiary and quaternary structure as other members of the CNG family, composed of four identical subunits.

**Fig. 1:**
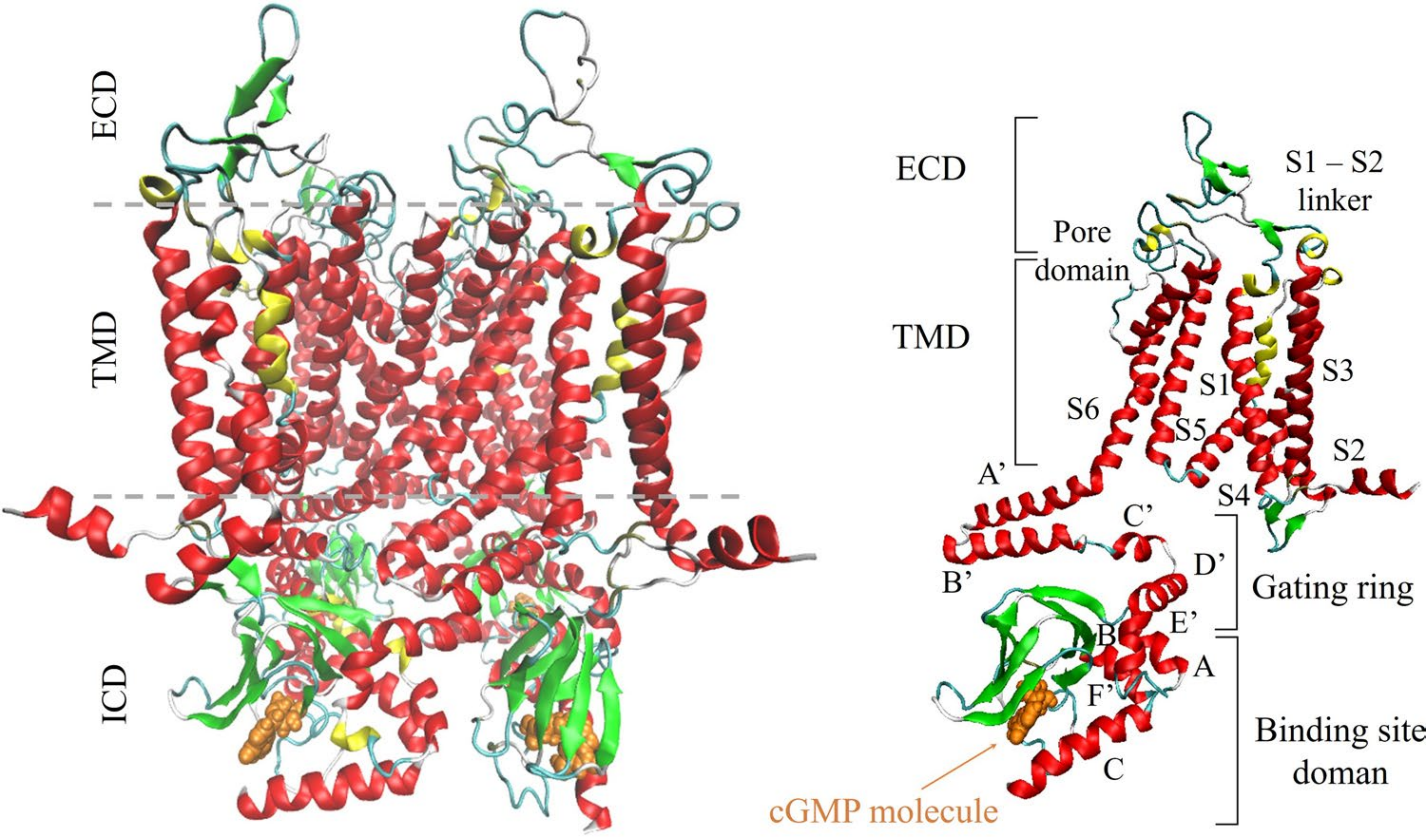
Structure of cGMP-bound WT TAX-4 channel (PDB ID 6WEK Zheng et al. 2020), in cartoon representation. In the left panel, the extracellular (ECD) and intracellular (ICD) boundaries are indicated by dashed lines. In the right panel, details of the subunit A’s extracellular and intracellular domains are shown. The cyclic guanosine monophosphate molecule is in orange vdW representation. Different colors in the protein identify secondary structure elements. The structural visualization is made with the Visual Molecular Dynamics (VMD) software v. 1.9.3 (Humphrey et al. 1996)

The tetramer, in the case of TAX-4 a homotetramer, builds up a channel with a transmembrane domain (TMD), an intracellular domain (ICD), and an extracellular domain (ECD) (see Fig. 1, left panel). Each subunit contains six helices (S1−S6), a pore loop between S5 and S6, and a cytoplasmic C-linker immediately following S6. The C-linker contains six $\alpha$ helices, named A’-F’; helices A’-D’ form the gating ring. This region is often collectively referred to as the C-linker/gating ring. The binding pocket (the binding site domain, BSD) is in the ICD, directly connected to the gating ring (Fig. 1, right panel). In CNG channels, the binding of cGMP molecules in the BSD pockets activates the channel, inducing the conformational change from the inactive into the open conductive channel form. Experimentally, the closure of CNG depends directly on a decrease in cGMP concentration, with a consequent reduction in the cytoplasmic calcium concentration (Zagotta and Siegelbaum 1996; Kaupp and Seifert 2002; Matulef and Zagotta 2003; Dai and Varnum 2013; Mazzolini et al. 2018; Young et al. 2001; Wang et al. 2007).

The conduction of Na^+^ and Ca^2+^ is the main functional role of TAX-4; therefore, the identification of structural determinants of conductivity is of relevance both from the point of view of electrophysiology and of disease characterization, in the perspective of pharmacological development.

In this respect, the recent Cryo-EM determination of the complete structure of CNG TAX-4 in both the unliganded closed-state and liganded open-state (Zheng et al. 2020; Li et al. 2017) represents a major advancement in the study of this channel. As a first step toward a complete identification of the permeation mechanisms, in this work we investigate the structural and functional properties of the wild-type CNG TAX-4 channel via molecular dynamics simulations of the full-length protein embedded in a lipid bilayer/water system. Simulations are performed both in the presence of cGMP molecules and in the unbound conformation, on the microsecond time scale.

Several channel structural descriptors are examined, and a first-level functional annotation is carried out. Results are compared with available experimental data for TAX-4 (Zheng et al. 2020; Li et al. 2017) and its human homologues (Hu et al. 2023; Hu and Yang 2023; Zheng et al. 2022). We identify the bound form as the pre-open conformation and the unbound form as the closed conformation of the TAX-4 channel, respectively. The comparison between the two modeled conformations, in turn, enables us to suggest the main changes involved in the binding-to-gating process.

## Materials and methods

### Models

We rely on the Cryo-EM structure of wild type (WT), cGMP-bound channel TAX-4 (Zheng et al. 2020) (PDB ID 6WEK) of *C. elegans*. The structure of the bound state corresponds to the amino acidic sequence 103−620. Missing residues (161−166) within the investigated sequence are modeled via CHARMM-GUI web-based graphical user interface (Jo et al. 2008; Wu et al. 2014). Missing residues 1−102, corresponding to a helix and coiled region before the S1 helix, are not modeled. The amino acidic sequence of each subunit is capped by acetyl and amide at N- and C-termini. The systems are prepared by evaluating the amino acids’ protonation state at pH 7.4, using the ProteinPrepare tool of the Play Molecule web interface (Martínez-Rosell et al. 2017). There are two non-protonated glutamates at the entrance of the selectivity filter, described below in detail. We use CHARMM-GUI to reconstruct the protein environment in a lipid membrane (1-palmitoyl-2-oleoyl-sn-glycero-3-phosphocholine, POPC), and the entire system is put in a box containing water and a standard concentration (0.150 M) of salt solution. We set a 15 Å thick water layer over and under the POPC membrane and an initial length of 180 Å in the X and Y directions.

**Fig. 2:**
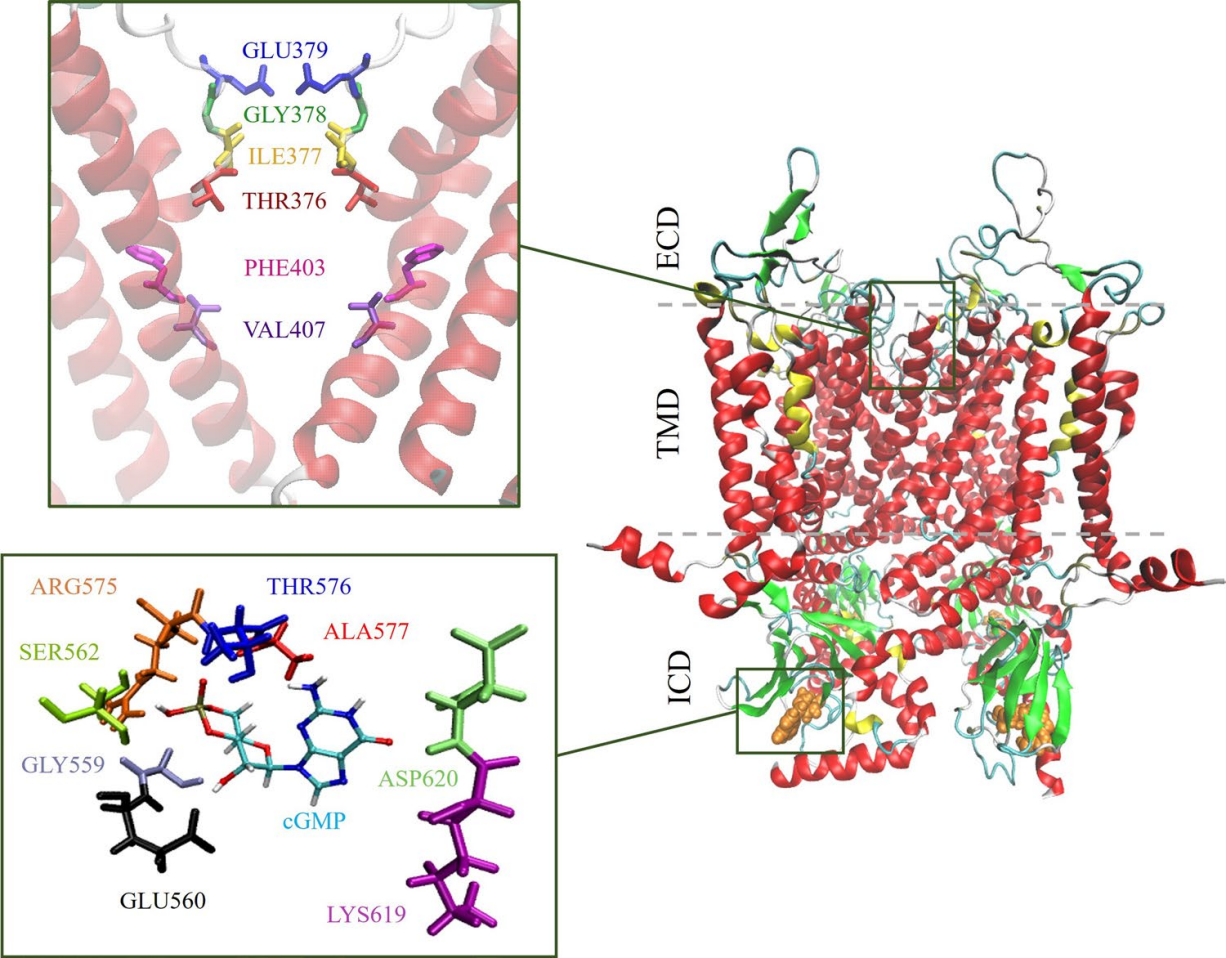
Definition of selectivity filter/central cavity and binding site domain within the bound-state TAX-4 channel. The selectivity filter is situated at the entrance of the channel pore on the extracellular side and is defined by four amino acids. The binding site domain is found on the cytosolic side of the channel and is characterized by eight amino acids

The BSD amino acids selection for the bound-state structure is based on a comparison among different organisms (Kaupp and Seifert 2002; Tibbs et al. 1998; Scott et al. 1996; Scott and Tanaka 1998; Altenhofen et al. 1991). The list includes GLY559, GLU560, SER562, ARG575, THR576, ALA577, LYS619 and ASP620. The selectivity filter/central cavity (SF/CC) (Li et al. 2017; Zheng et al. 2022, 2020) is composed of THR376, ILE377, GLY378, GLU379, PHE403, and VAL407. Visual representations of modeled SF/CC and BSD in the open bound-state model are shown in Fig. 2. The last two amino acids of the CC are responsible for the opening/closing states of the channel (Zheng et al. 2020); they define the so-called cavity gate (Dai 2022). A gating mechanism, conserved among different CNG channels, governed by a single central cavity gate involving two hydrophobic residues, has been proposed also in the homomeric CNGA1, heteromeric CNGA1/B1 rod channel, and the cone CNGA3/B3 channel, whose structures have been recently solved in the ligand-bound open and apo closed states (Zheng et al. 2022; Barret et al. 2022; Xue et al. 2021, 2022; Hu et al. 2023).

Starting from the WT open cryo-EM structure, we simulate two different systems:

i) open, cGMP-bound, in 0.150 M NaCl solution; the system is composed of 34549 atoms for solute (protein and ligands), 748 POPC molecules, 495 Na^+^ and 568 Cl^-^ ions, and 103008 water molecules;
ii) unbound, in 0.150 M NaCl solution; to build the unbound model, we start from the open cryo-EM structure removing the cGMP molecules. The system is composed of 34409 atoms for solute (protein), 748 POPC molecules, 496 Na^+^ and 569 Cl^-^ ions, and 103006 water molecules.

We use the Amber19SB force field (Tian et al. 2020) for protein and ions, together with the TIP3P water model (Jorgensen et al. 1983) and the sLipids force field (Jämbeck and Lyubartsev 2012a, b) for lipids. The cGMP parametrization was the one in Amber’s GAFF2 force field (He et al. 2020).

### Simulation protocols

We perform all-atom MD simulations with GROMACS software v. 2022.3 (Bekker et al. 1993; Berendsen et al. 1995). We minimize the input structure obtained from CHARMM-GUI with the steepest descent (Debye 1909; Haug et al. 1976) and conjugate gradient (Hestenes and Stiefel 1952; Nocedal and Wright 1999) algorithms in series, with a force tolerance of 10 kJ/mol$\cdot$nm. We then perform an equilibration phase in the NVT ensemble (using a V-rescale thermostat (Bussi et al. 2007) with $\tau$_T_ = 1 ps and T_ref_ = 300 K), with a time step of 1 fs, to relax the solvent around the protein and the membrane at different steps, changing the restraints’ strength (backbone and lateral chain) as follows:

1. 4$\times$10^3^ kJ/mol$\cdot$nm^2^ on protein and ligand backbone, 4$\times$10^3^ kJ/mol$\cdot$nm^2^ on protein side chains, 10^3^ kJ/mol$\cdot$nm^2^ on lipid backbone and dihedrals, at 300 K for 1 ns;
2. 2$\times$10^3^ kJ/mol$\cdot$nm^2^ on protein and ligand backbone, 2$\times$10^3^ kJ/mol$\cdot$nm^2^ on protein side chains, 5$\cdot$10^2^ kJ/mol$\cdot$nm^2^ on lipid backbone and dihedrals, at 300 K for 1 ns;
3. 5$\times$10^2^ kJ/mol$\cdot$nm^2^ on protein backbone, 2$\cdot$10^2^ kJ/mol$\cdot$nm^2^ on protein side chains, 2$\cdot$10^2^ kJ/mol$\cdot$nm^2^ on lipid backbone and dihedrals, at 300 K for 1 ns;
4. 2$\cdot$10^2^ kJ/mol$\cdot$nm^2^ on protein and ligand backbone, 50 kJ/mol$\cdot$nm^2^ on protein side chains, 50 kJ/mol$\cdot$nm^2^ on lipid backbone and dihedrals, at 300 K for 1 ns.

At the end of the equilibration phase, all restraints are removed for performing all the subsequent simulations. We perform a 2 ns simulated annealing run with T from 0 to 300 K, in the NVT ensemble. We include hydrogen bond constraints to rigid holonomic constraints using the LINCS algorithm with continuation (Hess et al. 1997). All simulations are performed at zero external electric fields in the NpT ensemble, using a V-rescale thermostat with $\tau$_T_ = 1 ps and T_ref_ = 300 K, and a semi-isotropic Parrinello-Rahman barostat (Parrinello and Rahman 1981) with $\tau$_p_ = 5 ps, compressibility values of 4.5$\times$10^-5^ bar^-1^ and p_ref_ = 1.0 bar. A 2 fs integration time step is set in all simulations. We use the particle mesh Ewald (Darden et al. 1993) technique for the long-range electrostatic interactions, with fourth-order cubic interpolation and 0.16 nm grid spacing for fast Fourier transform. The cutoff radius is 1 nm for electrostatic and van der Waals interactions, with an update of the neighbor list every 10 fs.

Our investigation includes i) three trajectories replicas, of 1 $\mu$s each, of the cGMP-bound channel in NaCl solution; the starting configurations of replicas 2 and 3 were picked along the replica 1 trajectory at different times (800 ns and 900 ns, respectively); ii) a 1.5 $\mu$s trajectory of the unbound channel in NaCl solution; the starting protein structure for this run was identical to the starting one of the bound run, where we removed the four ligands in the BSDs. This simulation has then been continued for an additional 1.5 $\mu$s, starting from the stationary unbound state reached at the end of the first run. Overall, the TAX-4 structure was assessed in simulation for a cumulative time of 6 $\mu$s.

### Analyses

We use PLUMED v. 2.9.0 (Bonomi et al. 2009; Tribello et al. 2014) for analyzing the MD trajectories. Protein structural stability is measured via i) the root mean square displacement (RMSD) of the channel from the equilibrated structure taken as reference (within the package tool gmx rms Maiorov and Crippen 1995), ii) the gyration radius of the channel (within the package tool gmx gyrate), and iii) the root mean square fluctuations (RMSFs) of amino acids from the equilibrated structure (within the package tool gmx rmsf). The reference structure is the configuration after the simulated annealing in the canonical ensemble without restraints. Binding modes of ligands are evaluated by the distribution of hydrogen bonds (within the package tool gmx hbonds Van der Spoel et al. 2006) between the ligands and the amino acids of the binding site domain (BSD), for each subunit. The channel functional annotation is conducted by analyzing the TMD pore structure and hydration. This is performed by measuring i) crossed distances between the COMs of the SF/CC amino acids of opposed subunits (A−C, B−D, respectively); crossed distances between selected atom pairs of the SF/CC amino acids are measured as well in the diagonally opposed A−C subunits; ii) the SF/CC pore radius profile, calculated with the HOLE v. 2.2.005 program (Smart et al. 1996). As water can be used as a proxy to predict hydrophobic gates (Klesse et al. 2019), the time evolution of the number of water molecules in the SF/IP channel region is monitored along all trajectories.

Snapshots are extracted every 1 ps along all equilibrium MD trajectories for statistical analyses. Both structural assessment (RMSD, gyration radius, COMs crossed distances), ligand binding modes and water count evaluation are performed over all the dynamics; RMSFs, pore radius profile and crossed distances between selected atom pairs in diagonally opposed subunits are calculated on representative structures, averaged over the last 200 ns of each trajectory.

## Results and discussions

In what follows, we present results for the cGMP-bound and unbound conformations, including the characterization of the full-length channel stability and details on the ligand binding modes. Subsequently, we present the results of the pore structure and hydration analysis. The comparison of the results obtained for the two structures allows us to shed light on the initial tertiary conformational changes following ligand uptake/release.

### The cGMP-bound state

#### Structure stability assessment

**Fig. 3:**
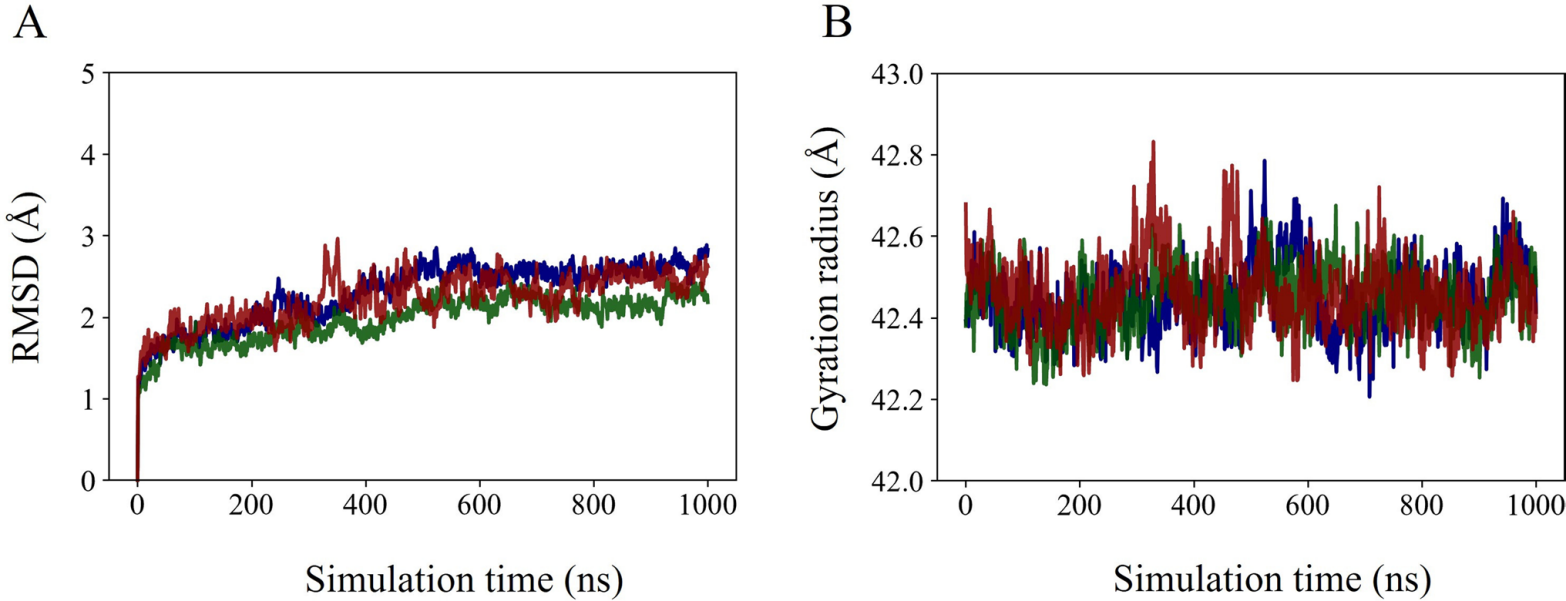
**A** Time evolution of the RMSD (in Å) of $\text {C}_{\alpha }$ atoms; **B** time evolution of the $\text {C}_{\alpha }$ atoms gyration radius (in Å). The equilibrated structure is chosen as the reference structure for the analysis, as defined in Section Analyses. Distinct colors identify replicas: replica 1, blue curve; replica 2, green curve; replica 3, red curve

As a preliminary characterization, in Fig. 3 we show the $\text {C}_{\alpha }$ atoms RMSD (panel A) and the gyration radius (panel B) time evolutions, along the three replicas trajectories. Very flat and low profiles around 2-3 Å are observed in the RMSD plot in each replica. Equilibrium structures are characterized by the average values of RMSD and gyration radius reported in Table 1. The three replica structures are equivalent, and they all undergo small relaxation with respect to the equilibrated structure used as reference.

**Table 1:** Average $\text {C}_{\alpha }$ atoms RMSD and gyration radius over the last 500 ns of simulation, for the three replicas

	RMSD (Å)	Gyration radius (Å)
Replica 1	2.58 ± 0.10	42.47 ± 0.09
Replica 2	2.19 ± 0.11	42.46 ± 0.07
Replica 3	2.41 ± 0.17	42.45 ± 0.07

**Fig. 4:**
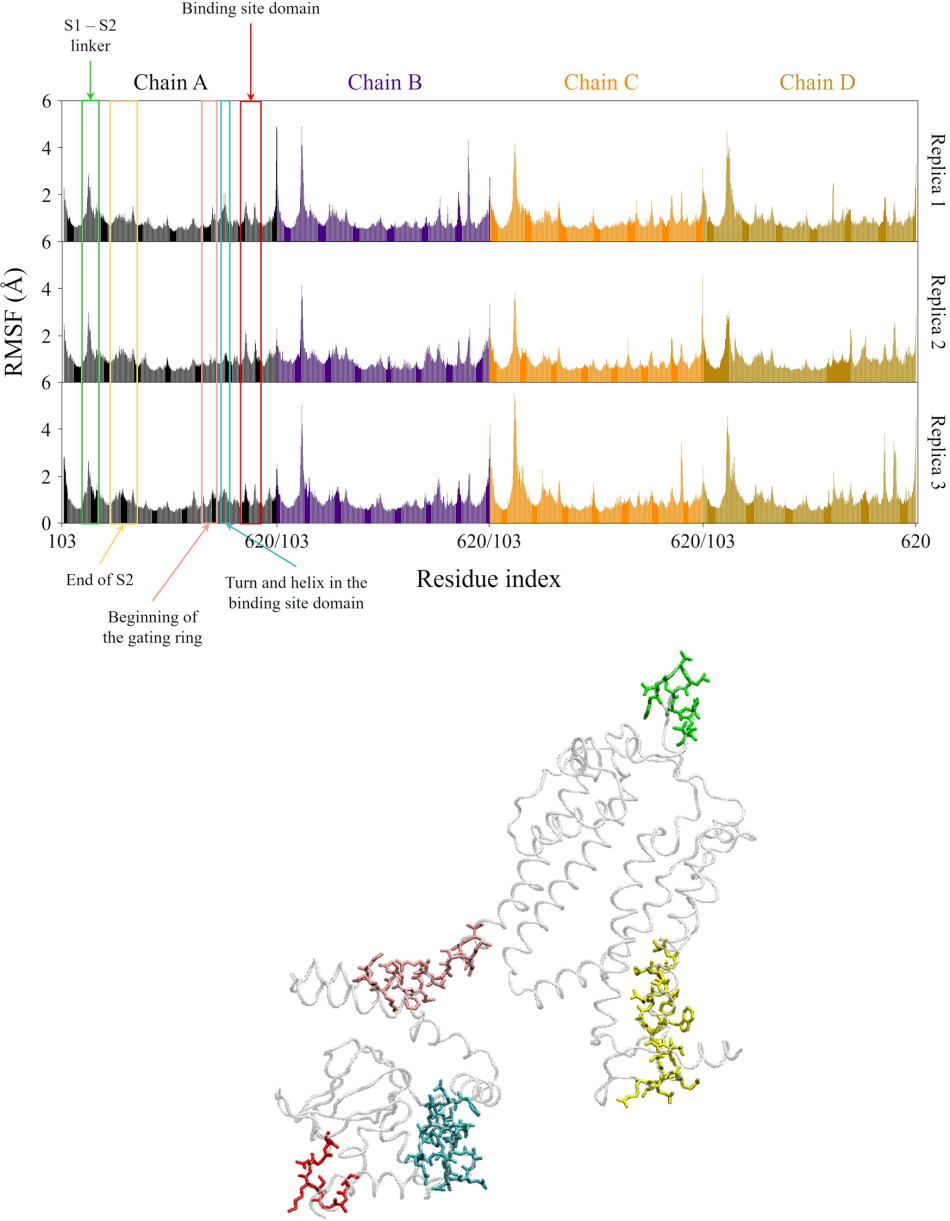
Time-averaged RMSFs distributions (in Å) of $\text {C}_{\alpha }$ atoms over the last 200 ns of each replica simulation. The A subunit structure with the highest flexible parts identification is also shown. The equilibrated structure, as defined in Section Analyses, is chosen as the reference structure for the analysis. Each subunit is identified by one color

In Fig. 4, we show the time-averaged RMS fluctuations (RMSFs) of $\text {C}_{\alpha }$ atoms computed along the last 200 ns portion of each replica trajectory, with respect to the equilibrated structure defined in Section Analyses. In the lower panel of Fig. 4 the highest peaks in the RMSFs plot are mapped onto the A subunit structure. The fluctuations are largely below 2 Å, except for five distinct regions with high mobility. Two of them are, as expected, the C- and N-termini of the subunit; the other two most flexible parts identified by the RMSFs plot are the turn within the S1−S2 linker (highlighted in green) and the turn within the binding site domain (in red). The S1−S2 linker peak is evident for almost all subunits. However, together with the BSD peaks, values are larger in both the B−D subunits, in particular along replicas 2 and 3.

#### Ligand-protein interaction

For the bound state model, we first evaluate the total number of hydrogen bonds between the ligands and the BSD residues.

**Fig. 5:**
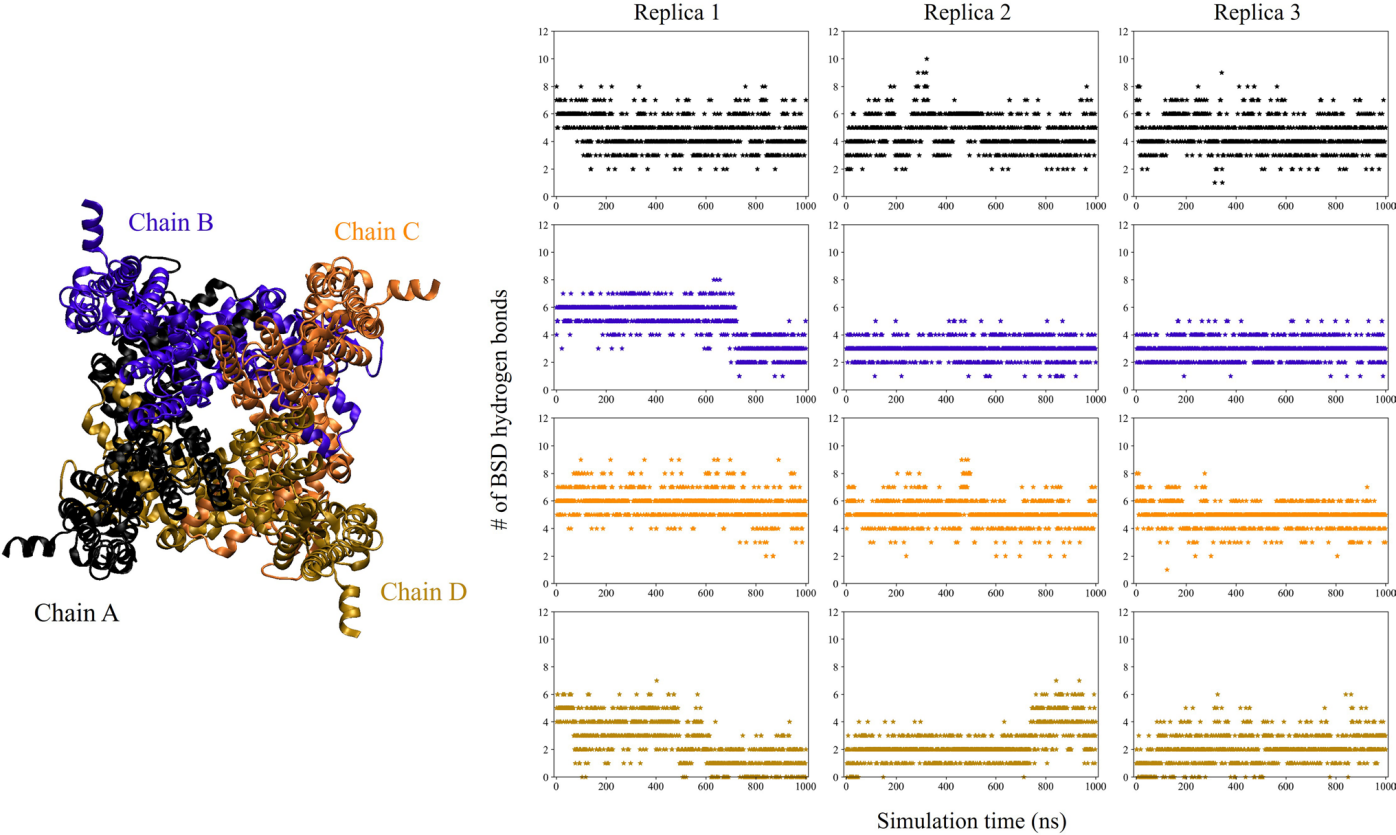
Time evolution of the total number of BSD hydrogen bonds, in the three replica simulations. Different colors identify the subunits, as highlighted by the top view of the channel on the left

For the three replicas, the time evolution of the hydrogen bonds within the BSD is reported in Fig. 5. The number of hydrogen bonds fluctuates along the simulation in the range 2-6, with some differences among the four subunits. Indeed, the diagonally opposed B−D chains are involved in a lower number of hydrogen bonds than the A−C chains, in particular along the replica trajectories 2 and 3. This behavior could be related to the larger RMSF peaks observed in the BSD in the subunits B−D.

Details on the BSD-cGMP hydrogen bonds are then carefully examined. In the following, a bond is identified by the lowercase letter that indicates if the atom is located in the backbone (m = main) or the side chain (s = side), followed by the name of the atom, i.e. sO1−mN. The first atom of the bond is related to the ligand, and the latter to the amino acid. Atom identification in the cGMP molecule is reported in Fig. S1 of Supplementary material. For each detected hydrogen bond, we evaluate the persistence over the trajectory. Results are fully reported in Fig. S2 of Supplementary material.

**Fig. 6:**
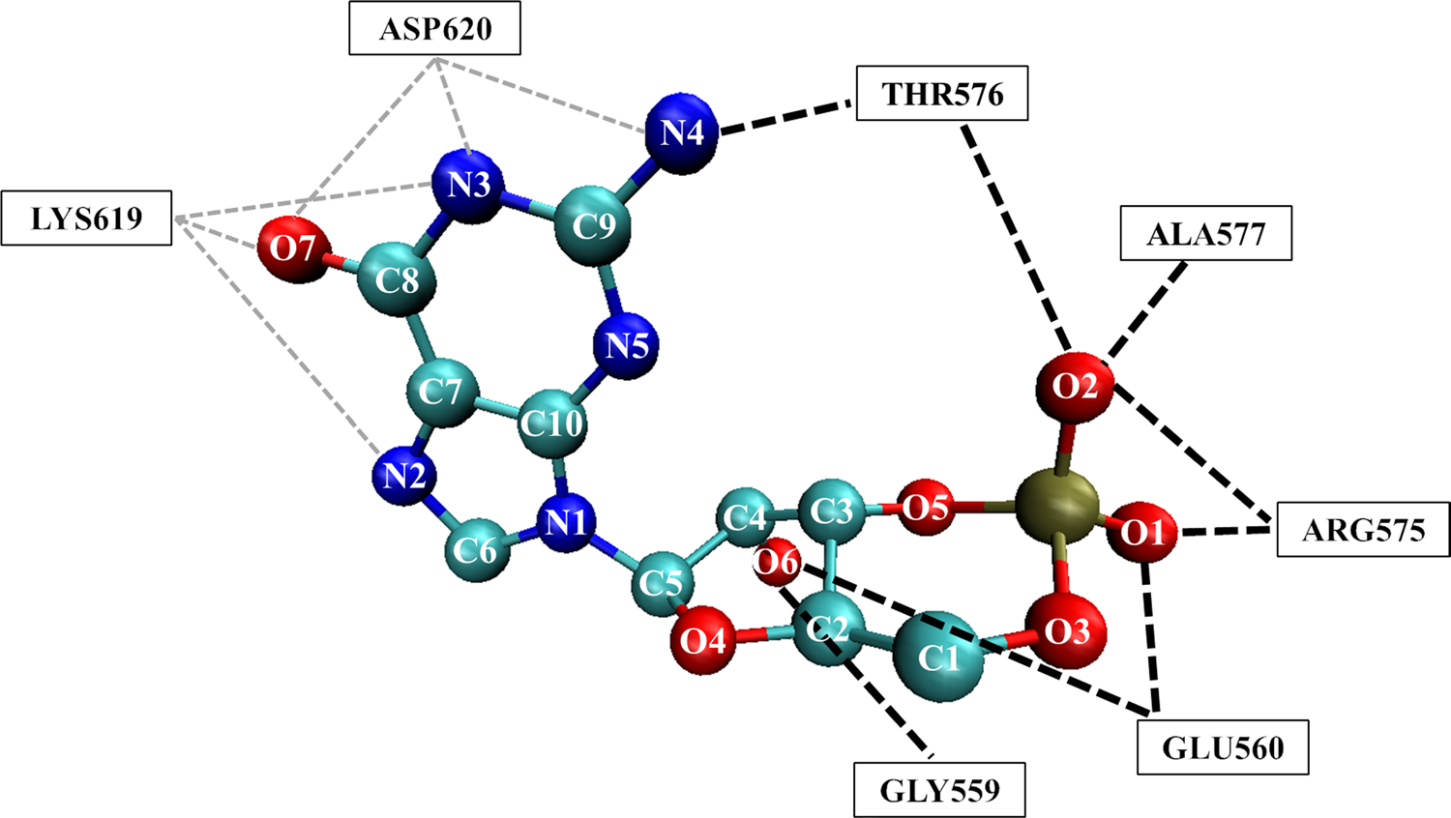
Main protein-ligand hydrogen bonds detected over the trajectories. Black dashed lines indicate bonds with persistence percentage > 10%; gray dashed lines indicate bonds with persistence percentage < 10%

It is notable that, except for SER562, the selected amino acids in the BSD form bonds with the ligand, in all chains, although with different persistence. In addition, chain B exhibits the lowest number of hydrogen bonds. At variance with the other subunits, bonds with ASP620 are never detected. Chain D is the subunit with less persistent BSD−ligand hydrogen bonds. The longeval hydrogen bonds are with i) GLY559, ii) GLU560, and iii) THR576, followed by ARG575 and ALA577. As shown in Fig. 6, GLY559 and GLU560 mostly interact with the pentose O6 oxygen atom, while ARG575 and ALA577 interact with the cGMP phosphate oxygen atoms. THR576 persistently bridges the cGMP phosphate O2 atom and the guanine nitrogen atom N4. To a minor extent, LYS619 interacts with the guanine oxygen O7 while ASP620 interacts with the guanine ring nitrogen atoms. Predicted BSD bonds agree with experimental findings on CNGs from other organisms (Scott et al. 2000; Kaupp and Seifert 2002; Scott et al. 1996; Scott and Tanaka 1998; Varnum et al. 1995; Tibbs et al. 1998; Altenhofen et al. 1991), in particular the interaction of THR576 with both the two moieties of cGMP is one of the most documented in the literature.

Besides the BSD amino acids defined in this work, other amino acids in chain D can form hydrogen bonds with the ligand (data not shown); in particular, these amino acids are GLU615, ILE616 (also reported in Scott et al. (1996)), LYS618 (found in all replicas), and LEU561 (detected in replicas 2 and 3).

To summarize, the hydrogen bond analysis conducted shows that the simulated structure is stably bound to the four ligand molecules, although in this respect the diagonally opposed subunits behave differently.

#### Pore structure characterization

**Fig. 7:**
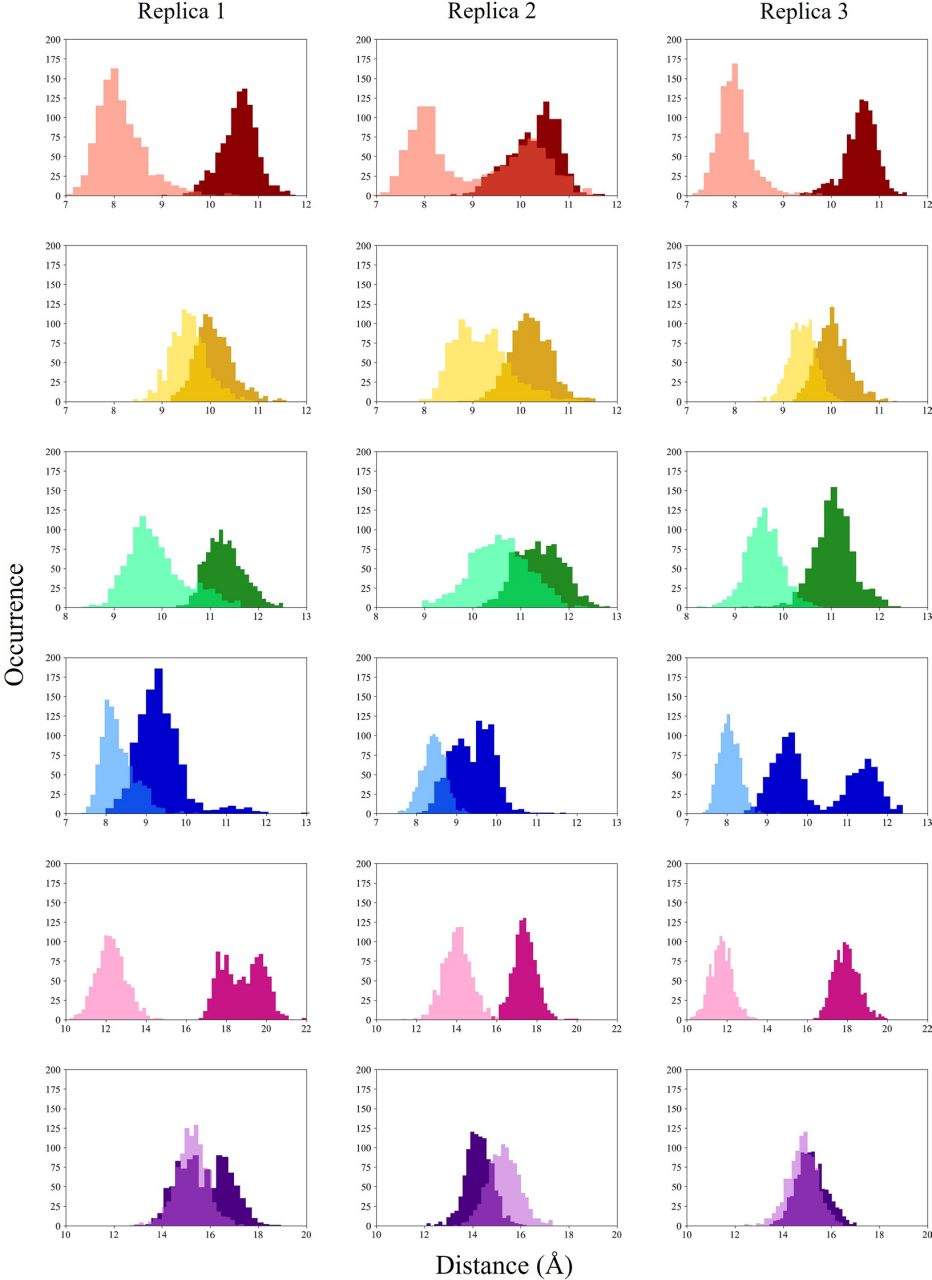
COM distances distributions for the six SF/CC amino acids. Dark colors indicate the A−C couple, while light colors indicate the B−D couple. Red: THR376; yellow: ILE377; green: GLY378; blue: GLU379; pink: PHE403; purple: VAL407

The distributions of the distances between the COMs of SF/CC amino acids in opposed subunits, evaluated over the trajectories, are shown in Fig. 7. Overall, peak values of the distributions could quantify the degree of opening in the bound conformation (Chiodo et al. 2015, 2018). Table 2 reports the crossed distances measured on representative bound and unbound conformations. These structures are averaged over the last 200 ns of the unbound and replica 3 bound trajectories, respectively. For comparison, we also report the corresponding values in the two TAX-4 experimental structures and the closed, pre-open, and open human homologues CNGA3/CNGB3 experimental structures.

**Table 2:** Crossed distances (in Å) between SF/CC COM of A−C couple (up) and B−D couple (down), in our models and the experimental cGMP-bound (PDB entry: 6WEK) and unbound (PDB entry: 6WEJ) TAX-4 structures, and open (PDB entry: 8EVC), pre-open (PDB entry: 8EVB), and closed (PDB entry: 8EV8) CNGA3/CNGB3 structures

A−C chains	THR376	ILE377	GLY378	GLU379	PHE403	VAL407
Unbound model (this work)	9.92	10.39	11.45	9.92	11.84	10.20
Unbound TAX-4 (Zheng et al. 2020)	11.47	10.36	11.76	9.31	11.04	9.25
Closed CNGA3/CNGB3 (Hu et al. 2023)	11.30	9.85	10.85	9.22	13.61	9.00
Pre-open CNGA3/CNGB3 (Hu et al. 2023)	11.01	9.43	10.38	10.14	15.88	11.50
Bound model (this work)	10.49	10.10	11.16	9.39	18.11	15.34
Open TAX-4 (Zheng et al. 2020)	11.51	10.14	11.79	9.31	21.01	16.53
Open CNGA3/CNGB3 (Hu et al. 2023)	11.15	9.65	10.75	9.22	21.21	15.40

B−D chains	THR376	ILE377	GLY378	GLU379	PHE403	VAL407
Unbound model (this work)	8.71	9.44	10.60	8.71	10.41	7.90
Unbound TAX-4 (Zheng et al. 2020)	11.28	10.26	11.59	9.16	10.89	9.34
Closed CNGA3/CNGB3 (Hu et al. 2023)	10.53	9.48	10.50	9.32	9.61	8.11
Pre-open CNGA3/CNGB3 (Hu et al. 2023)	10.54	9.47	9.54	9.10	12.88	15.90
Bound model (this work)	8.10	9.37	9.68	8.10	11.69	14.58
Open TAX-4 (Zheng et al. 2020)	11.64	10.24	11.90	9.31	21.16	16.92
Open CNGA3/CNGB3 (Hu et al. 2023)	10.73	9.37	10.49	9.05	17.18	15.78

At variance with the experimental data, there is a strong difference between the couples of opposed subunits A−C and B−D in the simulated bound conformation. In particular, for the SF amino acids (THR376, ILE377, GLY378, GLU379), the crossed distances are in good agreement with the experimental data in the A−C couple, while they are lower than the experimental ones in the B−D couple.

As for the central cavity, the PHE403 crossed distances lie around 18 Å in the A−C and 11.7 Å in B−D, to be compared with 21 Å in the experimental TAX-4 open conformation. The VAL407 crossed distances are mostly around 15 Å  both in A−C and B−D, compared with 16.5$-$16.9 Å in the experimental TAX-4. However, the PHE403 and VAL407 crossed distances are sizably greater than in the case of the experimental closed and pre-open structures (see Table 2) in the A−C case. At variance, in the B−D couple, the same distances are intermediate between the closed and the pre-open experimental ones.

**Fig. 8:**
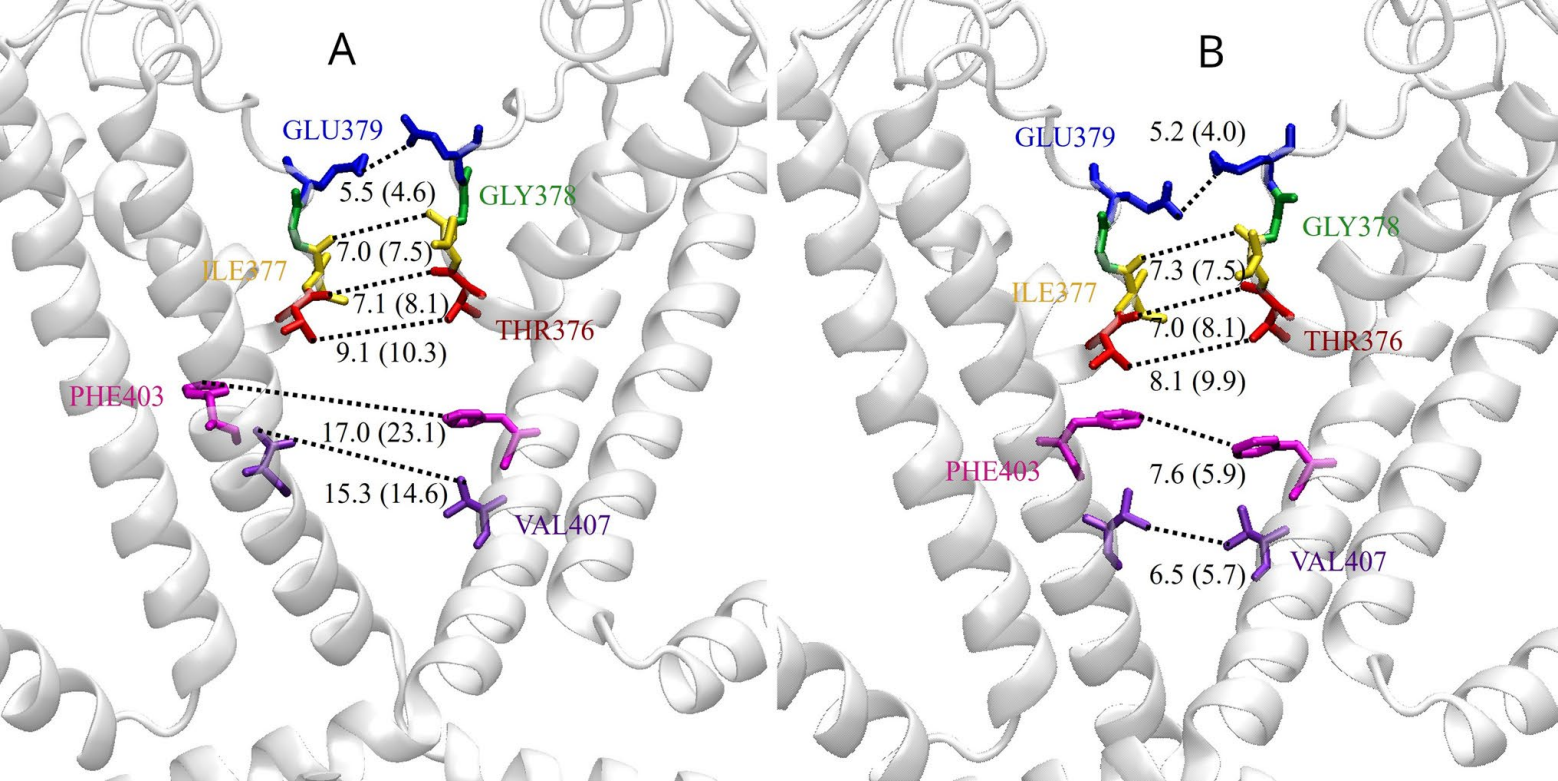
Distances (expressed in Å) of SF/CC amino acids in the A−C diagonally opposed subunits. Panel **A** cGMP-bound conformation. Distances are calculated within the structure averaged over the last 200 ns of the replica 3 trajectory. Panel **B** cGMP-unbound conformation. Distances are calculated within the structure averaged over the last 200 ns of the trajectory. Distance values (expressed in Å) of the experimental bound- and unbound structures (Zheng et al. 2020) are also reported for comparison

For the sake of direct comparison with the experimental findings, shown in Ref. Zheng et al. (2020), in Fig. 8 panel A distances between selected atom pairs of the A−C couple are reported on the average structure calculated over the last 200 ns of the replica 3. Analogous figures for replicas 1 and 2 are shown in the Supplementary material. Compared with the values in Ref. Zheng et al. (2020), these distances suggest that the simulated structure agrees well with the experimental open structure, at least in the SF region. The calculated CZ−CZ distances of PHE403 are $\sim$17 Å  in all replicas, to be compared with 23 Å  in the experimental open structure; the CG1−CG1 distances of VAL407 are in the range 13−15 Å  to be compared with 14.6 Å. These results suggest that the simulated structure is less open than the experimental one in the central cavity portion of the channel. Structural differences between MD-relaxed structures and their initial crystallographic templates are not uncommon in protein studies. As for ion channels, it has often been observed that relaxed structures in simulations exhibit narrower pores compared to experimentally determined ’wide-open’ ones (see e.g. Cerdan et al. (2018) for the glicine receptor, LeBard et al. (2012) for GLIC or Jia et al. (2018) for the BK channel). Indeed, dynamical fluctuations in pore width and hydration are to be expected during the simulation in a lipid/water/ions environment, unlike the static depiction provided by crystallography. Of note, in the realm of nicotinic receptors, a joint X-ray crystallography and simulation analysis of the GLIC, GluCL and GlyR receptors provided evidence in favor of a ‘semi-open’ conformation as being more representative of the active state in LGICs than the proposed GlyR ‘wide-open’ X-ray structure (Gonzalez-Gutierrez et al. 2017; Cerdan et al. 2018)).

**Fig. 9:**
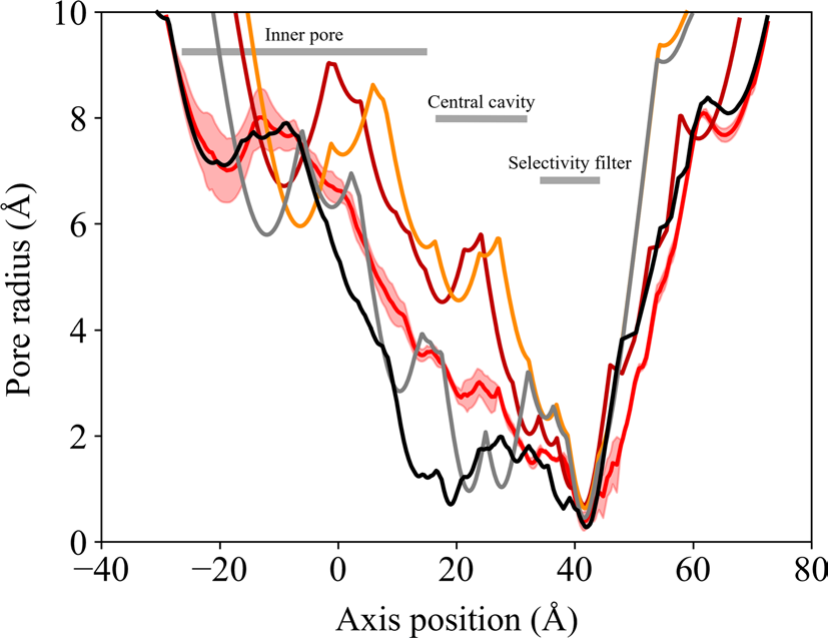
Pore profile evaluated with HOLE. The pore radius and channel axis distance are expressed in Å. Red curve: simulated bound conformation. The profile is the average of three profiles measured in the three replicas (on structures averaged over the last 200 ns of each trajectory). The shaded area reflects the standard deviation among the three simulations. Dark red and orange curves: experimental bound structures (PDB entry: 6WEK, 5H3O, respectively); gray curve: experimental unbound structure (PDB entry: 6WEJ) (Zheng et al. 2020; Li et al. 2017); dark curve: simulated unbound conformation (on the structure averaged over the last 200 ns of the trajectory)

Figure 9 shows the pore profile calculated with the HOLE tool on the bound structure. The profile is averaged over the three replica trajectories and compared to the profile in the experimental bound and unbound (closed) forms (Zheng et al. 2020). The minimum in all pore profiles is located at the entrance of the selectivity filter (GLU379).

As expected, the central cavity region displays a pore radius that is intermediate between those of the open and closed experimental structures. It is evident how the two bottlenecks present in the experimental unbound state (gray curve), located in correspondence to PHE403 and VAL407, are removed in the modeled bound conformation (red curve), as already suggested in Fig. 8. Several intermediate conformations between the open and the closed states have been experimentally identified also on the CNGA3/CNGB3 human homolog of TAX-4 (Hu et al. 2023; Hu and Yang 2023; Zheng et al. 2022). These structures are all fully cGMP-bound and their pore radius gradually increases to a value ranging between approximately 2-3 Å near the central cavity (Hu et al. 2023), similar to what is observed for the simulated bound TAX-4 structure. Gating intermediates are also found in other CNG channels, such as the human HCN1 (Burtscher et al. 2024) and the *Spirochaeta thermophila* SthK channel (Gao et al. 2022).

#### Pore hydration characterization

It is known that channel hydration observed in standard equilibrium simulations can be used as a reliable proxy for ion permeability in ion channels (Klesse et al. 2019). We analyze the channel hydration along the three replica trajectories to assess whether the cGMP-bound protein is in an open-like conductive conformation. Selected water molecules are identified as those occupying the region roughly from the top of the SF to the cytoplasmic end of S6. In the left panel of Fig. 10, we show the time series of the water count (replica 3); data relative to the other replicas are reported in the Supplementary material. A representative snapshot of the protein-water system is shown in the right panel of Fig. 10. The average number of water molecules is steadily fluctuating around 157, indicating that the channel is well-hydrated during the simulation. As shown in Fig. 10, the IP/CC pore is fully solvated and connected to the bulk water.

**Figure:**
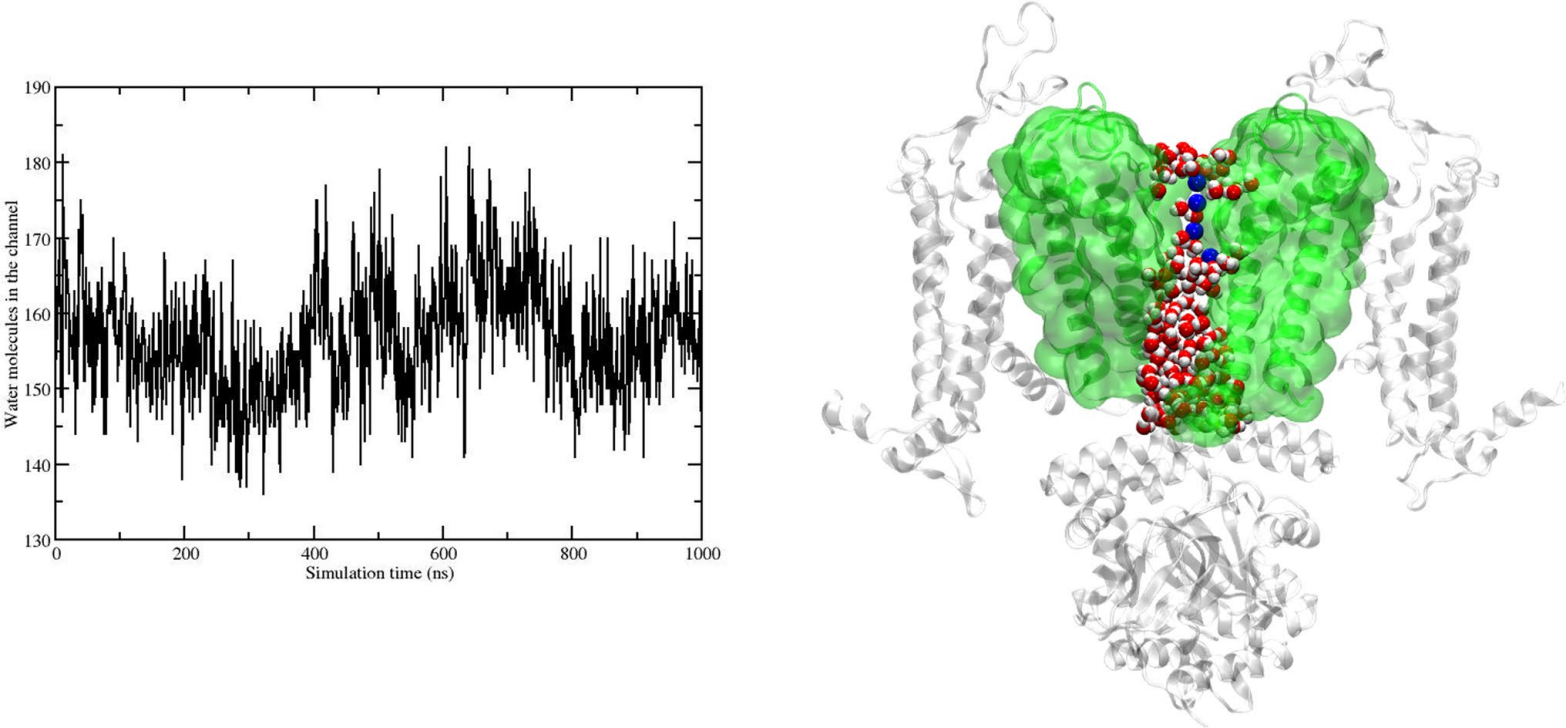
Left panel: Time series of the water molecules in the region from the top of the SF to the cytoplasmic end of S6; right panel: a representative snapshot of the protein structure represented in the cartoon representation, picked along the replica 3 trajectory. Only the diagonally opposed subunits A−C are represented, for visual clarity. S5, S6, and pore helices are highlighted in green and also shown in surface representation. The four persistent sodium ions are represented in vdW and colored in blue. Water in the channel is represented in vdW

Furthermore, few sodium ions are persistently present in time. Figure S4 shows the sodium ion probability isosurface averaged along the replica 3 trajectory. Similar results are found in the other replica trajectories (data not shown). We find three ions within the SF and another below the last SF amino acid (THR376). The presence of multiple ion-binding sites, formed by a combination of acidic side chains and backbone carbonyl oxygens, is a common feature of Ca^2+^-conducting channels. Our result agrees with the experimental findings of three sodium ions stably placed in the SF region of one of the two experimental open TAX-4 channels (Li et al. 2017; Xue et al. 2021, 2022).

We do not observe any ion permeation event in our cGMP-bound simulations. This is likely due to the fact that the time scale of the simulations is too short to observe any event in the absence of electric field, also given the known very low conductance of cGMP-activated channels (Savchenko et al. 1997). However, the simulated structure presents all the charateristics of an open-like conformation that could be exploited for future studies of ion permeability under electric fields.

### The cGMP-unbound state

#### Stability assessment

**Fig. 11:**
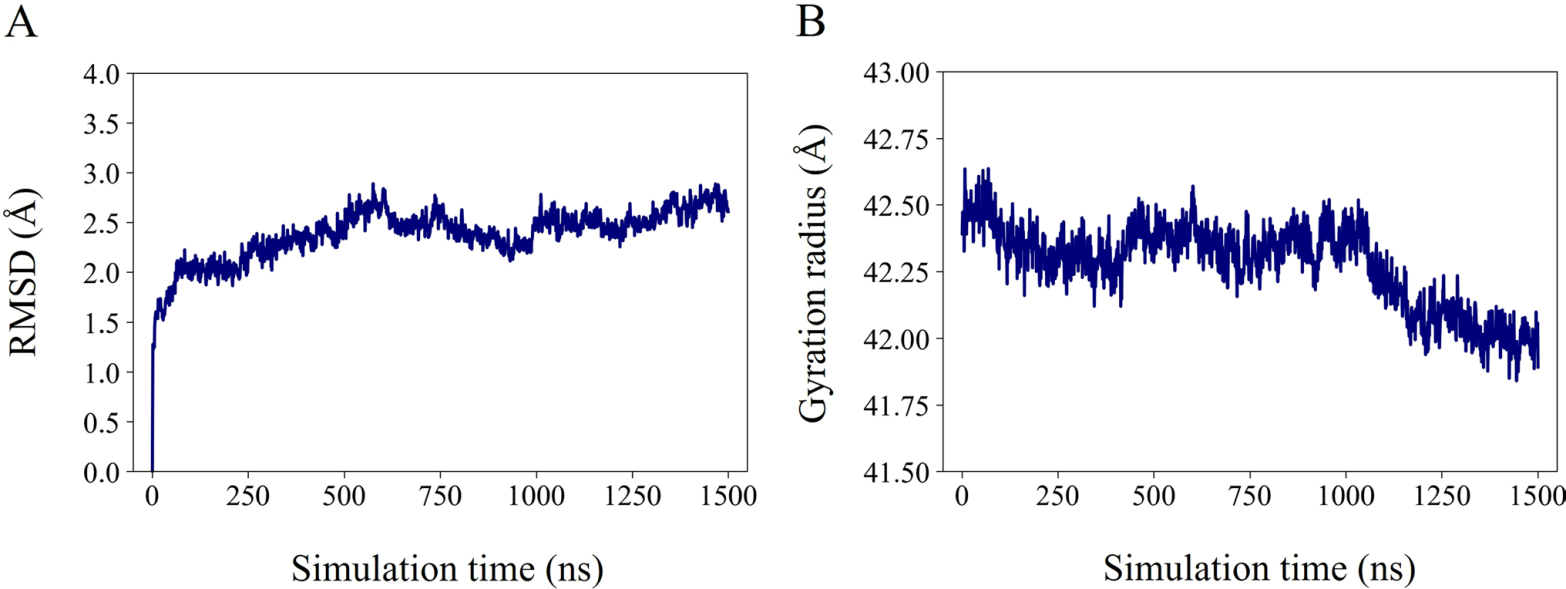
**A** RMSD (in Å) of protein $\text {C}_{\alpha }$ atoms over all the simulation; **B** gyration radius (in Å) over all the simulation. The equilibrated structure, as defined in Section Analyses, is chosen as the reference structure for the analysis

In Fig. 11, we show the RMSD evolution (panel A) and the gyration radius evolution (panel B) of protein $\text {C}_{\alpha }$ atoms over the simulation time.

In particular, there is a slight increase in the RMSD value starting from 1 $\mu$s, which coincides with a sizable decrease in the gyration radius, indicating that the protein is moving to a different conformation. As we shall see below, this is related to the closing mechanism of the pore. The RSMD and gyration radius averaged along the last 500 ns of the trajectory are 2.56±0.12 Å  and 42.11±0.11 Å, respectively.

**Fig. 12:**
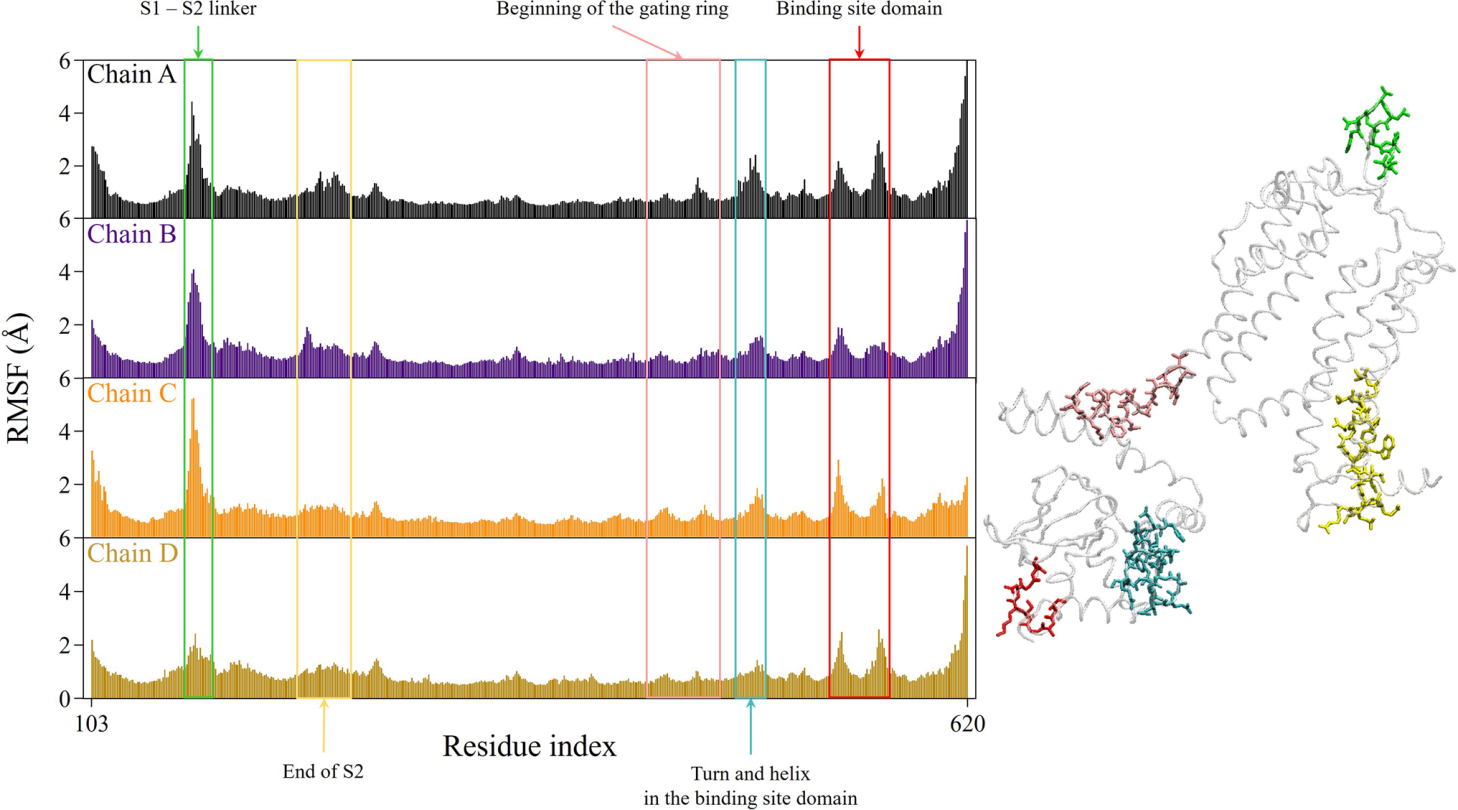
Time-averaged RMSF distributions (in Å) of $\text {C}_{\alpha }$ atoms over the last 200 ns of the simulation, and identification of flexible parts with different colors. The equilibrated structure, as defined in Section Analyses, is chosen as the reference structure for the analysis. Each subunit is identified by one color

Figure12 shows the RMSFs of the protein $\text {C}_{\alpha }$ atoms, computed along the last 200 ns of the trajectory, with reference to the equilibrated structure defined in Section Analyses. Fluctuations are largely below 2 Å  with some exceptions. The S1−S2 linker domain is one of the main peaks, with the lowest contribution for chain D. BSD is characterized again by two peaks, with a lower intensity for chain B. However, RMSFs in the BSD are overall larger than in the bound form, as expected given the absence of the ligands. The contribution of the C-terminus is quite absent for chain C.

#### Pore structure characterization

**Fig. 13:**
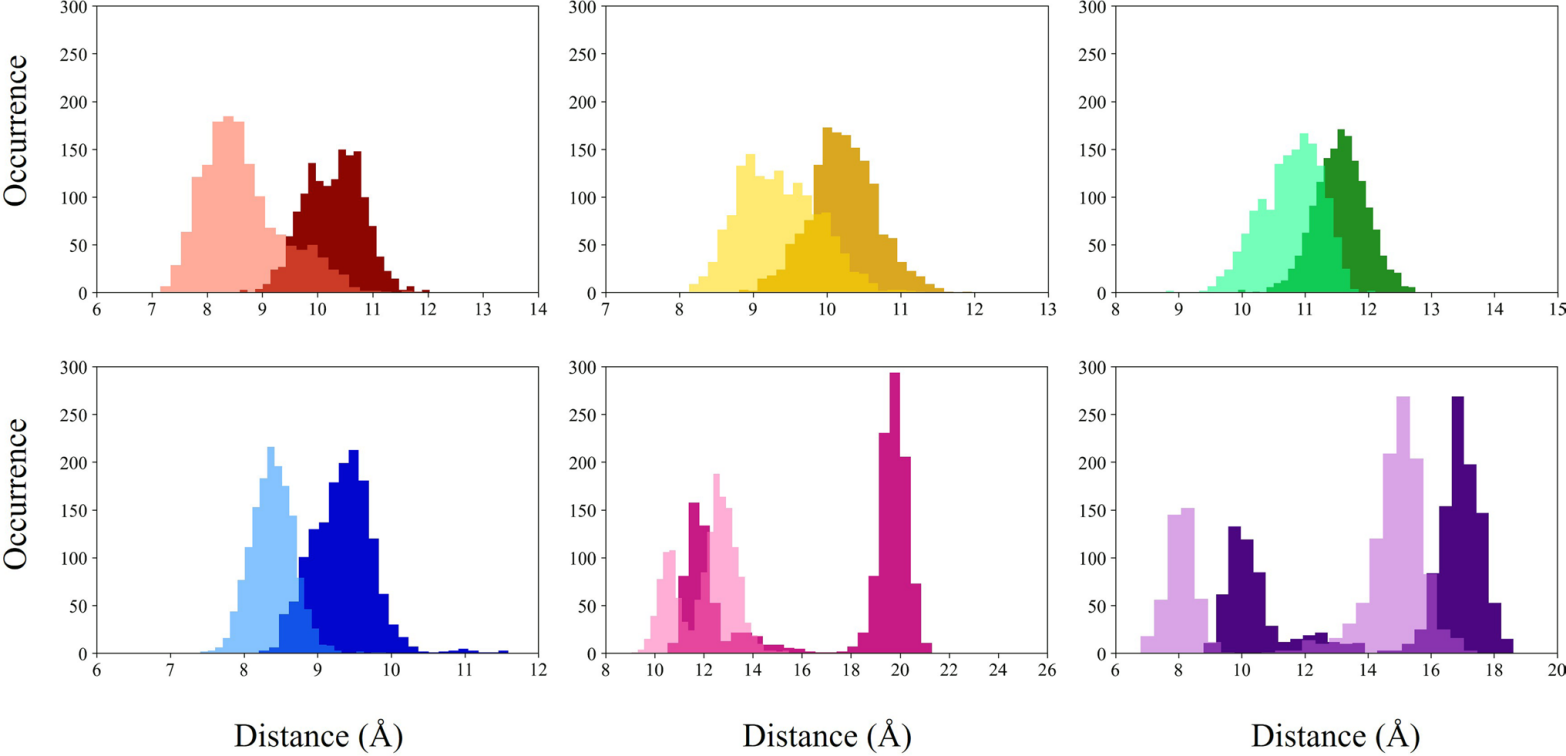
COM distance distributions (expressed in Å) for the six SF/CC amino acids. Dark colors indicate the A−C couple, while light colors indicate the B−D couple. Red: THR376; yellow: ILE377; green: GLY378; blue: GLU379; pink: PHE403; purple: VAL407

The crossed COM distances of SF/CC amino acids are shown in Fig. 13. Interestingly, PHE403 and VAL407 show a bimodal distribution, confirming that the protein visits two distinct conformations during the 1.5 $\mu$s trajectory. Indeed, the PHE403 distribution shows two main peaks at about 20 Å and 12 Å in the A−C couple. The VAL407 distance distribution shows two peaks at 17 Å  and 10 Å  in the A−C case and 15 Å  and 8 Å  in the B−D case.

As shown in Table 2, the PHE403 values measured on the structure averaged over the very last portion of the trajectory are 11.8 Å in A−C and 10.4 Å in B−D, to be compared with the value of 11 Å  in the experimental closed conformation of TAX-4 (see Table 2). As for VAL407, the average value is 10.2 Å in A−C and 9.3 Å in B−D to be compared with the experimental closed conformation value of 9.3 Å  in both opposite couples (see Table 2). These results overall suggest that in the final portion of the trajectory, the unbound structure is approaching a closed structure.

Similarly to the bound case, in Fig. 8 panel B we report the crossed distances between selected atom pairs in the SF/CC in subunits A and C, measured in the average structure over the last 200 ns of the unbound trajectory. The comparison with the same selected distances on the closed experimental structure of Ref. Zheng et al. (2020) further confirms that the unbound conformation is on average in a closed state. Furthermore, the comparison with the bound case shown in Fig. 8 panel A points out that the structure of the selectivity filter is not altered by the closing mechanism. The activation gate is thus entirely located lower down, in agreement with experimental data (Zheng et al. 2020).

The pore profile is shown in Fig. 9 (dark curve). Two minima are present, one in correspondence with the selectivity filter, as in the bound state; the other is located close to the cavity gate amino acids. As observed for the cGMP-bound conformation, also in the simulated unbound conformation the pore radius in the central cavity is lower compared to the experimental unbound structure.

#### Pore hydration characterization

**Fig. 14:**
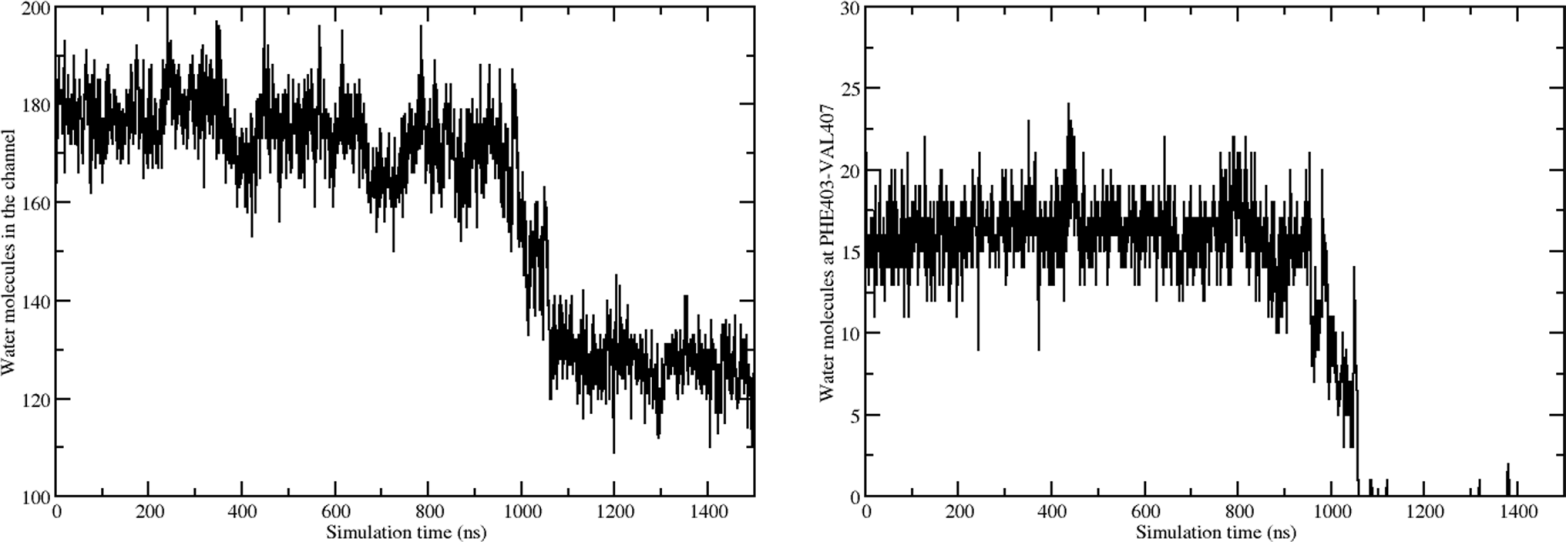
Left panel: time series of the water molecules in the channel; right panel: time series of the water molecules in the region comprised between PHE403 and VAL407

In Fig. 14 the time series of the water molecules inside the channel is shown along the first unbound trajectory. As already suggested by the RMSD and gyration radius plots, after approximately 1 $\mu$s something occurs within the channel. Overall, the average number of water molecules drops from a stationary value of 175 to 125 after about 1 $\mu$s, corresponding to a loss ($\Delta W$) of $\simeq$ 50 water molecules. A similar $\Delta W$ has been observed in the open-to-close transition of the BK channel (Jia et al. 2018) upon ligand removal, and in the dewetting transition of the closed hTRPV4 (Huang and Chen 2023).

In the region between PHE403 and VAL407, the water count drops from 15 to zero. The new channel hydration is stationary over the last $\sim$ 500 ns portion of the unbound trajectory. This behavior is further assessed based on an additional 1.5 $\mu$s simulation of the unbound conformation (data not shown), started from the end of the first run.

**Fig. 15:**
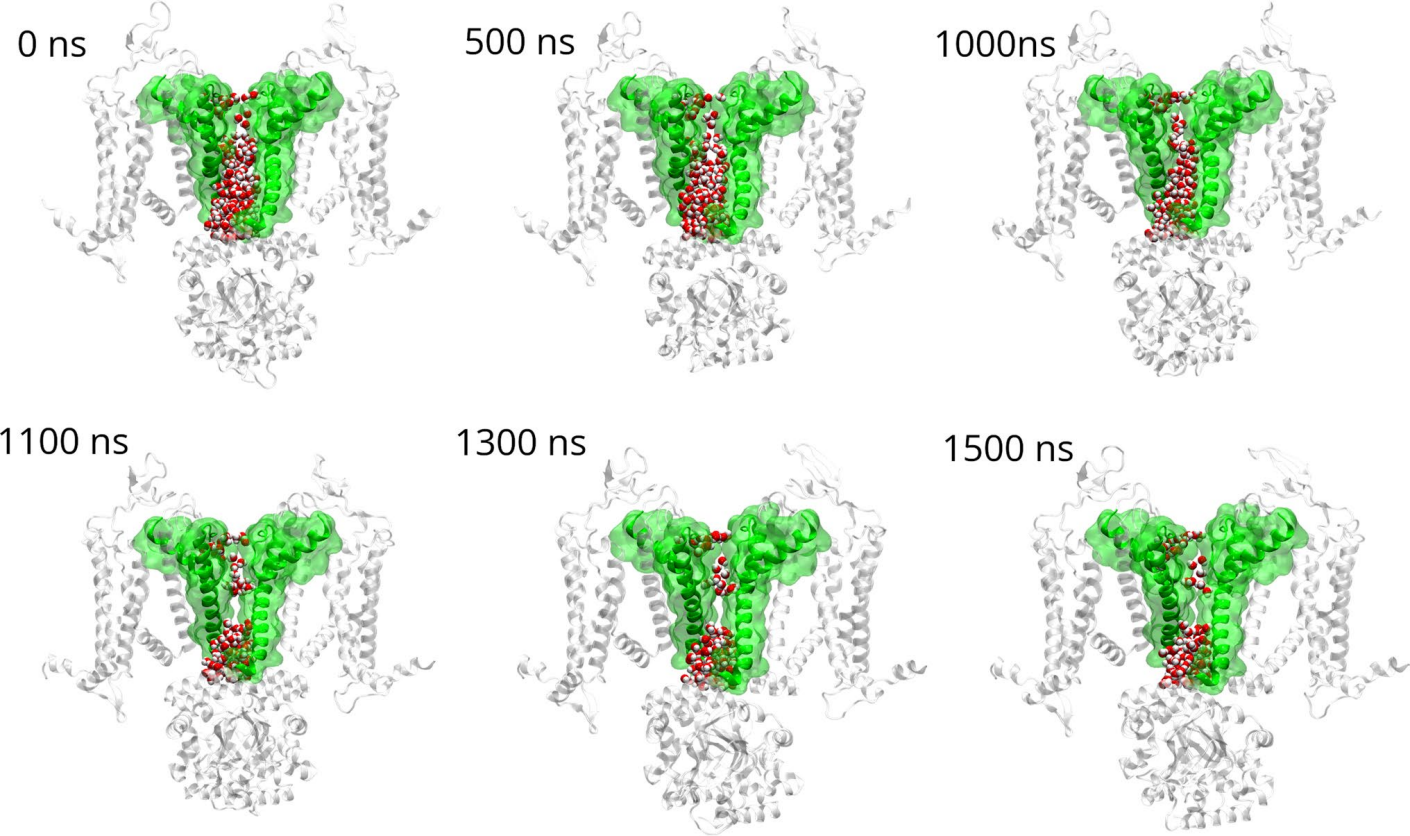
Snapshots of the protein structure, picked along the unbound trajectory. Only the diagonally opposed subunits A−C are represented, for visual clarity. The S6, S5, and pore helices are highlighted in green and shown both in cartoon and surface representation. Water in the channel is represented in vdW

Visual inspection of representative protein-water system snapshots in Fig. 15 confirms a gradual dewetting transition in the hydrophobic region PHE403−VAL407 along the trajectory.

**Fig. 16:**
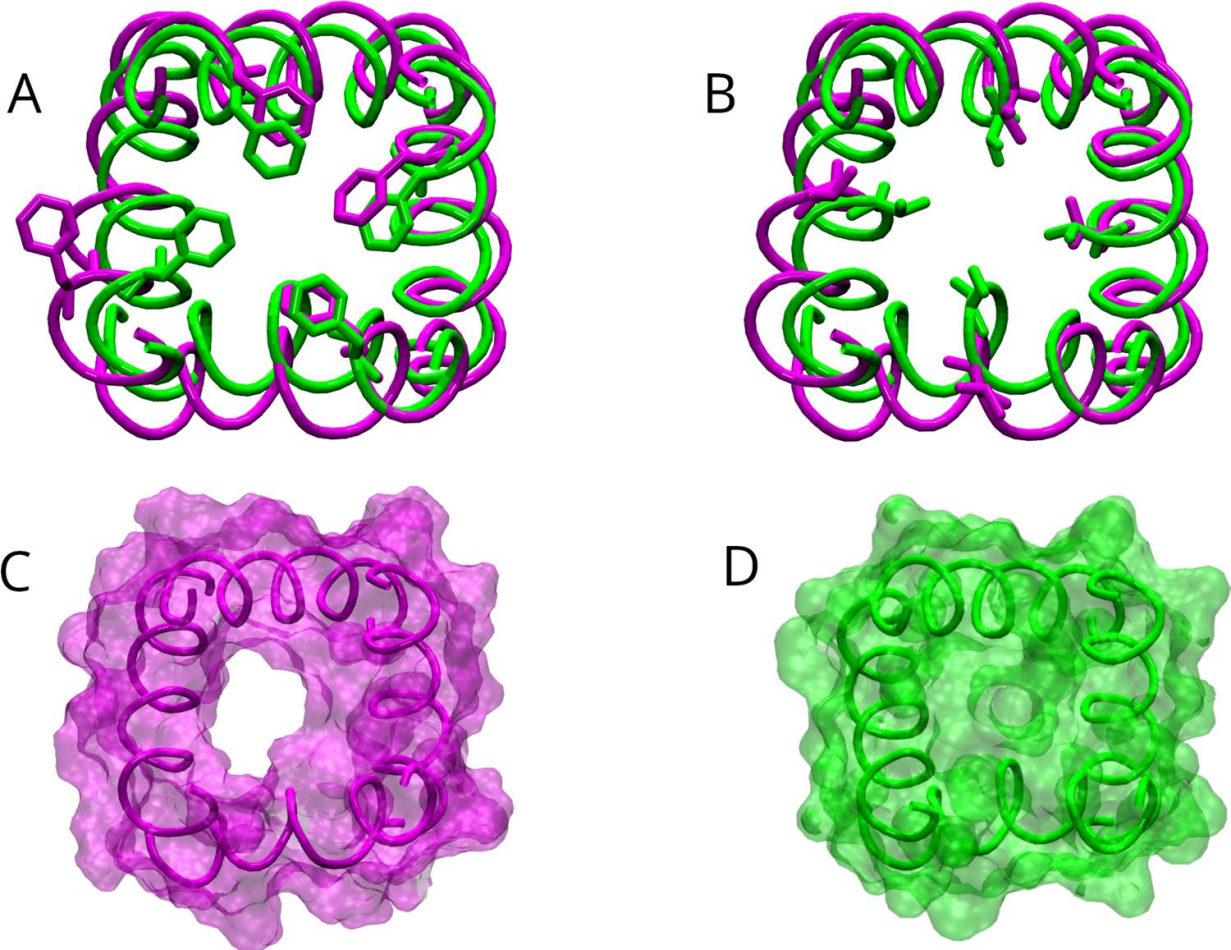
Comparison of the four S6 helices (region VAL400-SER415) as seen from the extracellular side, in the bound (replica 3, magenta) and unbound (green) structures averaged along the last 500 ns of each trajectory, highlighting the rotation of PHE403 (panel **A**) and VAL407 (panel **B**). The helices are shown in tube representation, and PHE403 and VAL407 are represented in licorice. The S6 helices are also shown in surface representation (panel **C**, bound; panel **D**, unbound), to highlight the presence/absence of an open pore. The two structures are superimposed based on the alignment of all $\hbox {C}_{\alpha }$ atoms (RMSD 2.5 Å)

To get insight into the relevant conformational changes leading to the channel dewetting, the four S6 helices in statistically representative (stationary) bound and unbound structures are superimposed and shown in Fig. 16, as seen from the extracellular side. The bound conformation exhibits an asymmetric behavior of the two couples of opposed subunits, where only one or at most two cavity gate residues (PHE403, VAL407) point away from the pore lumen. Compared to the experimental characterization of the CNGA3/CNGB3 human homolog (Hu et al. 2023; Hu and Yang 2023; Zheng et al. 2022), the bound conformation could be by analogy assigned as the “pre-open” intermediate of TAX-4. In this respect, the unbound configuration, where all four hydrophobic gate residues point toward the pore center, can be definitely assigned to the closed state of TAX-4.

The comparison between the bound and unbound conformation points out that dewetting is due to pore shrinking caused by the rotation inside the pore lumen of PHE403 in one out of four subunits, namely the A subunit (panel A), along with the corresponding rotation of two out of four VAL407 (panel B). Pore shrinking is better evidenced in the bottom panels of Fig. 16, where a surface representation of the same protein portions is also given in both the bound (panel C) and unbound (panel D) structures.

#### Binding-to gating transition in TAX-4

**Fig. 17:**
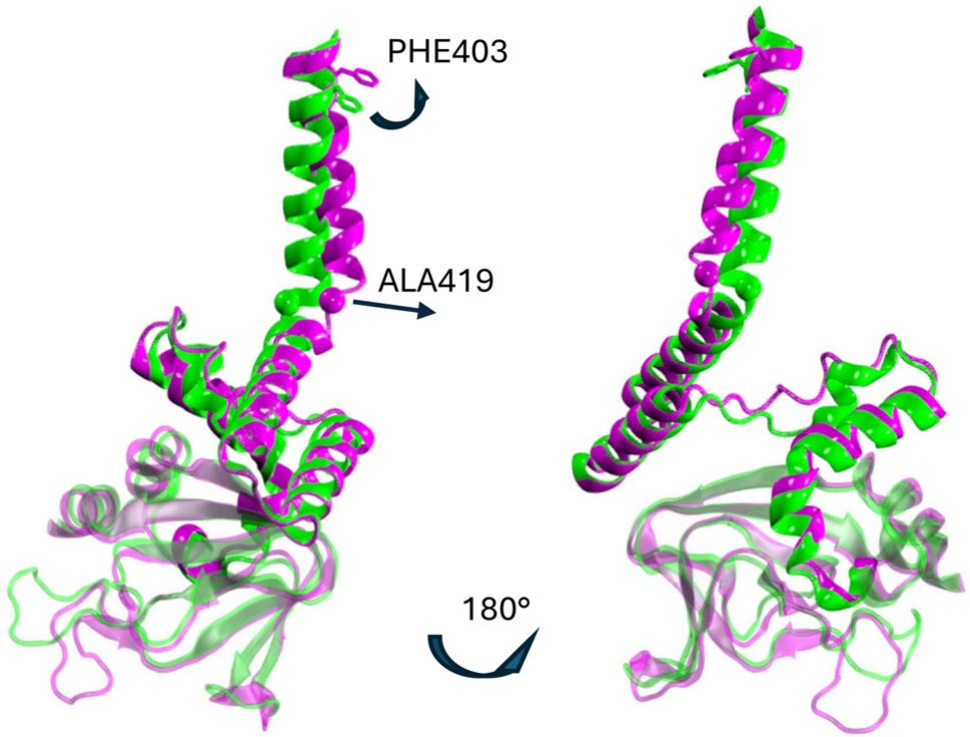
Superposition of the chain A (residues 400−600) in the bound (replica 3, magenta) and unbound (green) structures averaged along the last 500 ns of each trajectory, highlighting cGMP-induced conformational changes. Protein is represented in cartoon; PHE403 is represented in licorice; the $\text {C}_{\alpha }$ atom of ALA419 is represented in vdW. The two structures are superimposed based on the alignment of all $\text {C}_{\alpha }$ atoms (RMSD 2.6  Å)

The availability of two stable structures, one ligand-bound, open-like, and well-hydrated and one unbound closed, allows us to speculate on the possible changes involved in the binding-to-gating mechanism in the TAX-4 channel. To this end, chains A (residues 400−600) in the two protein conformations are superimposed (Fig. 17), based on the alignment of all protein $\text {C}_{\alpha }$ atoms (RMSD 2.6 Å). Apart from minor changes around the BSD $\beta$ roll, we observe an overall tilt of the C-linker helices in the bound with respect to the unbound conformation, most prominent in the gating ring helices A’B’ (see Fig. 1, right panel, for helix labeling). However, the main difference found is at the level of the A’ helix. Here a radial expansion of ALA419 ($\hbox {C}_{\alpha }$ unbound-bound distance = 5.0 Å) located at the beginning of the A’ helix is totally responsible for the S6 helix twist, which in turn induces the PHE403 (VAL407) swinging outside (inside) from the pore axis in the bound (unbound) conformation. The results agree with the findings obtained by comparing the two putative open and closed experimental states (Zheng et al. 2020; Li et al. 2017), i.e., the conformational changes induced by cGMP binding are effectively transmitted from the BSD to the cavity gate via the C-linker region.

## Conclusions

In this work, atomistic molecular dynamics simulations of the TAX-4 channel in both cGMP-bound and unbound conformations are conducted for the first time (according to the literature), using the recently resolved Cryo-EM structure of the open state of the full-length wild-type TAX-4 channel from *C. elegans*. Zheng et al. (2020).

Protein structural stability and ligand-binding modes are assessed on the microsecond time scale. Results suggest distinct behavior for the diagonally opposed subunits A−C and B−D in the TAX-4 tetramer. This asymmetry is evident both at the level of the SF/CC, where the B and D chains are closer to each other than the A and C chains and in the interactions with ligands.

Structural and functional analysis of the ion channel pore reveals an open-like, well-hydrated conformation in the presence of cGMP. Based on the comparison with the human homologues, we could assign this as the TAX-4 “pre-open” conformation, where chain asymmetry is a defining feature. Since pore hydration has been suggested as a proxy for ion permeability, in this work we consider this structure to be representative of the ‘active’ state, and suitable for starting studying ligand-induced activation mechanisms.

Upon ligand removal, channel closure occurs within about one microsecond. A hydrophobic gating mechanism is observed in TAX-4, similar to other ion channels (Huang and Chen 2023; Jia et al. 2018; Aryal et al. 2015, 2014; Trick et al. 2016; Beckstein and Sansom 2006; Yamashita et al. 2017; Zheng et al. 2018; Jensen et al. 2010; Chiodo et al. 2018; Yazdani et al. 2020; Rao et al. 2021; Guardiani et al. 2022), that involves the rotation of PHE403 and VAL407 within the pore lumen leading to the central cavity dewetting. This hydrophobic dewetting transition generates a dry pore stable on the microsecond timescale

Finally, the comparison between the modeled bound and unbound conformations enables us to speculate on key conformational changes underlying the binding-to-gating transition.

## Supplementary Information

Below is the link to the electronic supplementary material.

Supplementary file1 (PDF 2442 KB)

## Data Availability

The data supporting this study’s findings are available from the corresponding authors upon reasonable request.
