# Supplementary material for: Structural and functional characterization of Caenorhabditis elegans cyclic GMP-activated channel TAX-4 via molecular dynamics simulations
Source: Eur Biophys J. 2025 May 27;55(2):135–53. doi: 10.1007/s00249-025-01756-w (PMC13109178; doi:10.1007/s00249-025-01756-w)
Supplement: Supplementary file 1 — Supplementary file1 (PDF 2442 KB) [file 249_2025_1756_MOESM1_ESM.pdf]

Structural and Functional Characterization of *Caenorhabditis elegans* Cyclic GMP-activated Channel TAX-4 via Molecular Dynamics Simulations
Supplementary material

Nicole Luchetti[1,2], Marco Lauricella[3], Velia Minicozzi[4], Grazia Cottone[5*], Letizia Chiodo[1*]

[1]Department of Engineering, Università Campus Bio-Medico di Roma, Via Álvaro del Portillo 21, Rome, 00128, Italy.
[2]Centre for Life Nano- & Neuro-Science, Italian Institute of Technology, Viale Regina Elena 291, Rome, 00161, Italy.
[3]Istituto per le Applicazioni del Calcolo, Consiglio Nazionale delle Ricerche, Via dei Taurini 19, Rome, 00185, Italy.
[4]Department of Physics and INFN, "Tor Vergata" University, Via della Ricerca Scientifica 1, Rome, 00133, Italy.
[5]Department of Physics and Chemistry "Emilio Segrè", University of Palermo, Viale delle Scienze 17, Palermo, 90128, Italy.

*Corresponding author(s). E-mail(s): grazia.cottone@unipa.it; l.chiodo@unicampus.it;
Contributing authors: n.luchetti@unicampus.it; m.lauricella@iac.cnr.it; velia.minicozzi@roma2.infn.it;

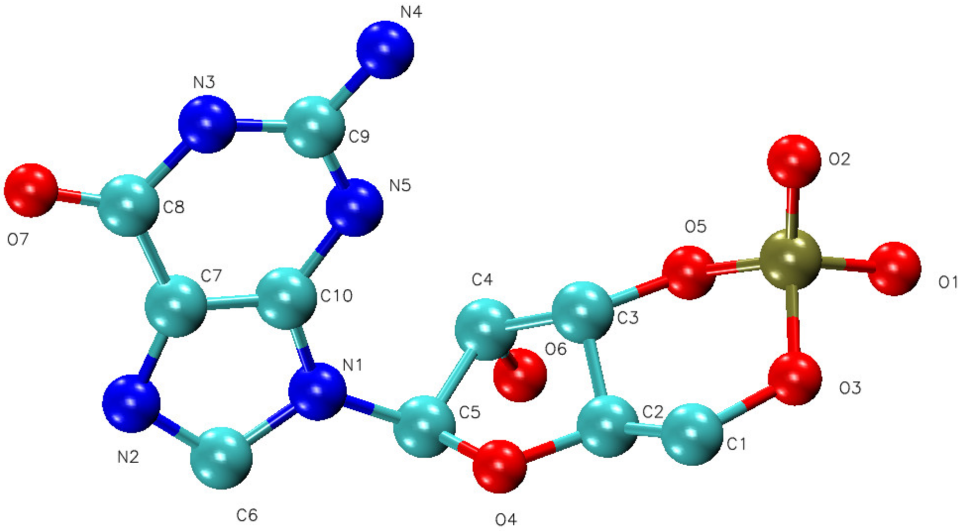

Fig. S1: Cyclic guanosine monophosphate in licorice representation with atom labeling. Hydrogen atoms are not represented.

Replica	Amino acid	Chain A	Chain B	Chain C	Chain D
Replica 1	GLY559	sO6 – mN (3.4)	**sO6 – mN (21.1)**	**sO6 – mN (34.5)**	sO3 – mN (0.2)
	GLU560	**sO6 – sOE1 (57.6)**, sO6 – sOE2 (6.6)	**sO6 – sOE1 (41.8)**, **sO6 – sOE2 (18.1)**	**sO6 – sOE1 (17.4)**, **sO6 – sOE2 (36.7)**	sO6 – sOE1 (1.1), sO6 – sOE2 (0.2), **sO1 – sOE1 (11.5)**, sO1 – sOE2 (7.1)
	SER562	–	–	–	–
	ARG575	sO3 – sNH1 (0.3), **sO1 – sNH1 (17.0)**	sO2 – sNH1 (8.7), sO1 – sNH1 (9.3)	sO1 – sNH1 (5.2)	sO1 – sNH1 (1.7)
	THR576	**sO2 – sOG1 (12.7)**, **sO2 – mN (22.6)**, sN4 – sOG1 (5.6)	**sO2 – sOG1 (22.2)**, **sO2 – mN (39.2)**, **sN4 – sOG1 (19.7)**, sN5 – sOG1 (0.1)	**sO2 – sOG1 (35.3)**, **sO2 – mN (58.2)**, **sN4 – sOG1 (32.6)**	sO2 – sOG1 (8.3), **sO2 – mN (15.9)**, sN4 – sOG1 (5.9), sN4 – mO (0.1)
	ALA577	**sO2 – mN (16.2)**	sO2 – mN (1.4)	sO2 – mN (0.3)	sO2 – mN (0.7)
	LYS619	sO7 – sNZ (3.6)	sO7 – sNZ (1.6)	sO7 – sNZ (3.2), sN3 – mO (0.1)	sO7 – sNZ (0.2), sN3 – mO (3.6), sN4 – mO (4.1)
	ASP620	sN4 – sOD1 (2.8), sN4 – sOD2 (3.9), sN3 – sOD1 (3.1), sN3 – sOD2 (2.7), sN4 – mO (0.4), sN3 – mO (0.3), sN4 – sOXT (0.1), sN3 – sOXT (0.9)	–	sN4 – sOD1 (4.2), sN4 – sOD2 (4.5), sN3 – sOD1 (0.5), sN3 – sOD2 (0.4), sN4 – mO (0.6), sN3 – mO (0.2), sN4 – sOXT (0.5), sN3 – sOXT (0.2)	sN4 – sOD1 (0.8), sN4 – sOD2 (0.2), sN3 – sOD1 (0.2), sN3 – sOD2 (0.1)
Replica 2	GLY559	sO6 – mN (7.7)	**sO6 – mN (10.1)**	**sO6 – mN (38.0)**	sO3 – mN (0.2), sO6 – mN (0.9)
	GLU560	**sO6 – sOE1 (42.6)**, **sO6 – sOE2 (16.3)**	**sO6 – sOE1 (39.6)**, **sO6 – sOE2 (25.7)**	**sO6 – sOE1 (27.1)**, **sO6 – sOE2 (30.4)**	**sO6 – sOE1 (18.0)**, sO6 – sOE2 (9.8), **sO1 – sOE1 (29.8)**, **sO1 – sOE2 (14.5)**
	SER562	–	–	–	–
	ARG575	sO3 – sNH1 (0.1), **sO1 – sNH1 (20.0)**	**sO1 – sNH1 (32.8)**	sO1 – sNH1 (0.5)	sO1 – sNH1 (1.0), sO3 – sNH1 (1.0)
	THR576	sO2 – sOG1 (4.6), **sO2 – mN (25.6)**, sN4 – sOG1 (4.6)	–	**sO2 – sOG1 (36.6)**, **sO2 – mN (46.3)**, **sN4 – sOG1 (27.6)**	sO2 – sOG1 (6.5), sO2 – mN (5.3), sN4 – sOG1 (4.6), sN4 – mO (0.1)
	ALA577	**sO2 – mN (12.2)**	–	sO2 – mN (0.3)	sO2 – mN (0.3)
	LYS619	sO7 – sNZ (2.6)	sO7 – sNZ (1.7), sN2 – sNZ (0.5)	sO7 – sNZ (2.4), sN3 – mO (0.1)	sO7 – sNZ (1.8), sN3 – mO (0.1), sN4 – mO (0.2)
	ASP620	sN4 – sOD1 (1.5), sN4 – sOD2 (3.1), sN3 – sOD1 (3.5), sN3 – sOD2 (2.0), sN4 – mO (0.3), sN3 – mO (1.4), sN4 – sOXT (0.6), sN3 – sOXT (0.9)	–	sN4 – sOD1 (3.1), sN4 – sOD2 (2.3), sN3 – sOD1 (1.5), sN3 – sOD2 (1.1), sN4 – mO (0.1), sN4 – sOXT (0.1)	sN4 – sOD1 (1.1), sN4 – sOD2 (0.2), sN3 – sOD1 (0.3), sN3 – sOD2 (0.1), sN3 – mO (0.1)
Replica 3	GLY559	sO6 – mN (1.8)	sO6 – mN (5.7)	**sO6 – mN (36.5)**	sO1 – mN (3.8), sO2 – mN (2.6), sO3 – mN (0.3)
	GLU560	**sO6 – sOE1 (51.9)**, **sO6 – sOE2 (10.6)**	**sO6 – sOE1 (45.7)**, **sO6 – sOE2 (23.7)**	**sO6 – sOE1 (25.8)**, **sO6 – sOE2 (37.4)**	sO6 – sOE1 (0.1), sO1 – sOE2 (8.8), **sO1 – sOE2 (50.6)**
	SER562	–	–	–	–
	ARG575	sO3 – sNH1 (0.4), **sO1 – sNH1 (15.7)**	**sO2 – sNH1 (34.0)**	sO3 – sNH1 (0.9)	–
	THR576	sO2 – sOG1 (4.1), **sO2 – mN (21.7)**, sN4 – sOG1 (0.9)	–	**sO2 – sOG1 (33.5)**, **sO2 – mN (45.6)**, **sN4 – sOG1 (23.1)**, sN4 – sOG1 (0.1)	sO2 – sOG1 (0.4), sO7 – sOG1 (2.3), sN4 – sOG1 (0.7), sN2 – sOG1 (1.2)
	ALA577	**sO2 – mN (15.7)**	–	sO2 – mN (0.2)	–
	LYS619	sO7 – sNZ (2.6)	sO7 – sNZ (3.5), sN2 – sNZ (0.6)	sO7 – sNZ (1.1), sN3 – mO (0.1), sN2 – sNZ (0.1)	sN3 – mO (1.4), sN4 – mO (0.8)
	ASP620	sN4 – sOD1 (2.7), sN4 – sOD2 (5.6), sN3 – sOD1 (6.7), sN3 – sOD2 (2.9), sN4 – mO (1.5), sN3 – mO (0.3), sN4 – sOXT (0.2), sN3 – sOXT (1.5)	–	sN4 – sOD1 (1.8), sN4 – sOD2 (1.7), sN3 – sOD1 (0.4), sN3 – mO (0.3), sN4 – sOXT (0.1), sN3 – sOXT (0.1), sN3 – mO (0.1)	sN4 – sOD1 (0.8), sN4 – sOD2 (1.2), sN3 – sOD1 (1.0), sN3 – mO (0.4), sN4 – sOXT (3.7), sN3 – sOXT (1.9), sN3 – mO (3.8), sN4 – sOD1 (2.0), sO7 – mN (0.5)

Fig. S2: Main hydrogen bonds and persistence over all the dynamics, expressed in percentage; bonds with persistence percentage > 10% are reported in bold. Hydrogen bonds include bonds between the side nitrogen and oxygen of cGMP and both backbone and side-chain nitrogen and oxygen of amino acids. A couple of first and second atoms indicate cGMP atom (as indicated in Figure S1) − a.a. atom. Missing bonds are identified by −. The notation is m = main and s = side.

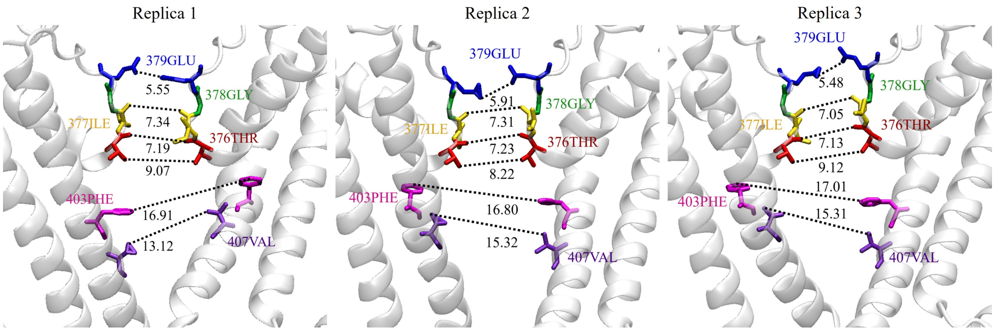

Fig. S3: Distances (in Å) of selectivity filter/central cavity amino acids atoms pairs in the A−C diagonally opposed subunits. Distances are calculated within the structure averaged over the last 200 ns of each replica trajectory.

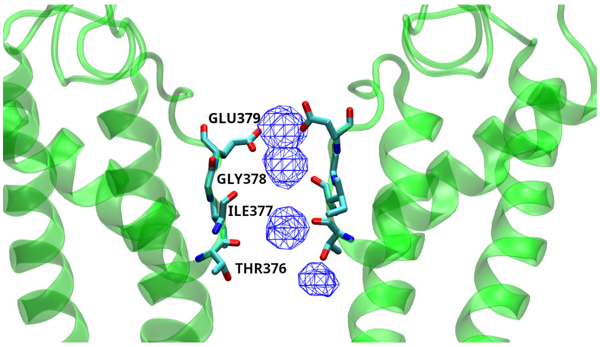

Fig. S4: Sodium probability density isosurface in the pore SF (replica 3). Isovalue: 0.01. The four residues of the SF are also highlighted in licorice representation.

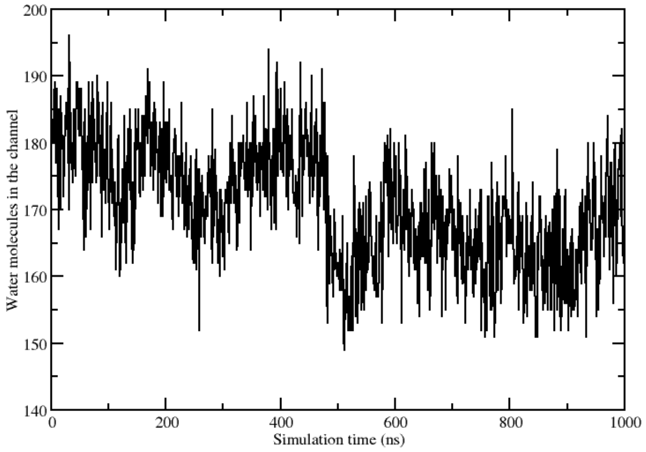

Fig. S5: Time series of the water molecules in the channel from the top of the SF to the cytoplasmic end of S6, in the replica 1 cGMP-bound trajectory.

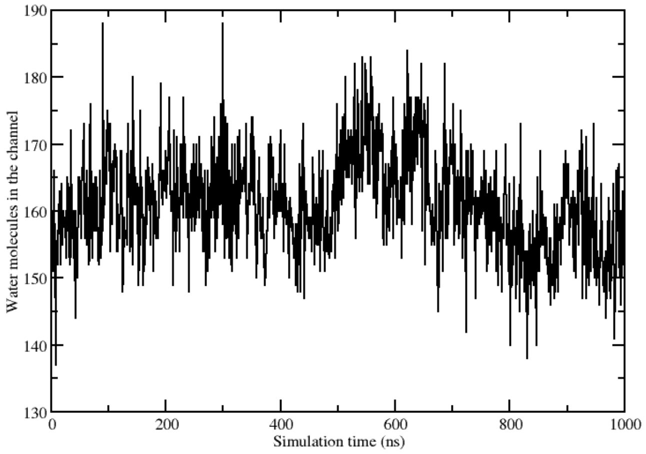

Fig. S6: As in Fig. S5, along the replica 2 cGMP-bound trajectory.
